# Harnessing immunotherapy for brain metastases: insights into tumor–brain microenvironment interactions and emerging treatment modalities

**DOI:** 10.1186/s13045-023-01518-1

**Published:** 2023-12-16

**Authors:** Dairan Zhou, Zhenyu Gong, Dejun Wu, Chao Ma, Lijun Hou, Xiaomin Niu, Tao Xu

**Affiliations:** 1 Department of Neurosurgery, Changzheng Hospital, Naval Medical University, 415 Fengyang Road, Huangpu District, Shanghai, 200003 People’s Republic of China; 2 Department of Neurosurgery, Klinikum Rechts Der Isar, Technical University of Munich, Munich, 81675 Germany; 3 Department of Neurosurgery, The Second Affiliated Hospital of Anhui Medical University, Hefei, 230601 Anhui People’s Republic of China; 4 Department of Shanghai Lung Cancer Center, Shanghai Chest Hospital, Shanghai Jiao Tong University School of Medicine, 241 Huaihai West Road, Xuhui District, Shanghai, 200030 People’s Republic of China

**Keywords:** Brain metastases, Tumor microenvironment, Immunotherapy, Immune checkpoint inhibitors, CAR-T cells, Glial cells

## Abstract

Brain metastases signify a deleterious milestone in the progression of several advanced cancers, predominantly originating from lung, breast and melanoma malignancies, with a median survival timeframe nearing six months. Existing therapeutic regimens yield suboptimal outcomes; however, burgeoning insights into the tumor microenvironment, particularly the immunosuppressive milieu engendered by tumor–brain interplay, posit immunotherapy as a promising avenue for ameliorating brain metastases. In this review, we meticulously delineate the research advancements concerning the microenvironment of brain metastases, striving to elucidate the panorama of their onset and evolution. We encapsulate three emergent immunotherapeutic strategies, namely immune checkpoint inhibition, chimeric antigen receptor (CAR) T cell transplantation and glial cell-targeted immunoenhancement. We underscore the imperative of aligning immunotherapy development with in-depth understanding of the tumor microenvironment and engendering innovative delivery platforms. Moreover, the integration with established or avant-garde physical methodologies and localized applications warrants consideration in the prevailing therapeutic schema.

## Introduction

### Epidemiology

**Fig. 1:**
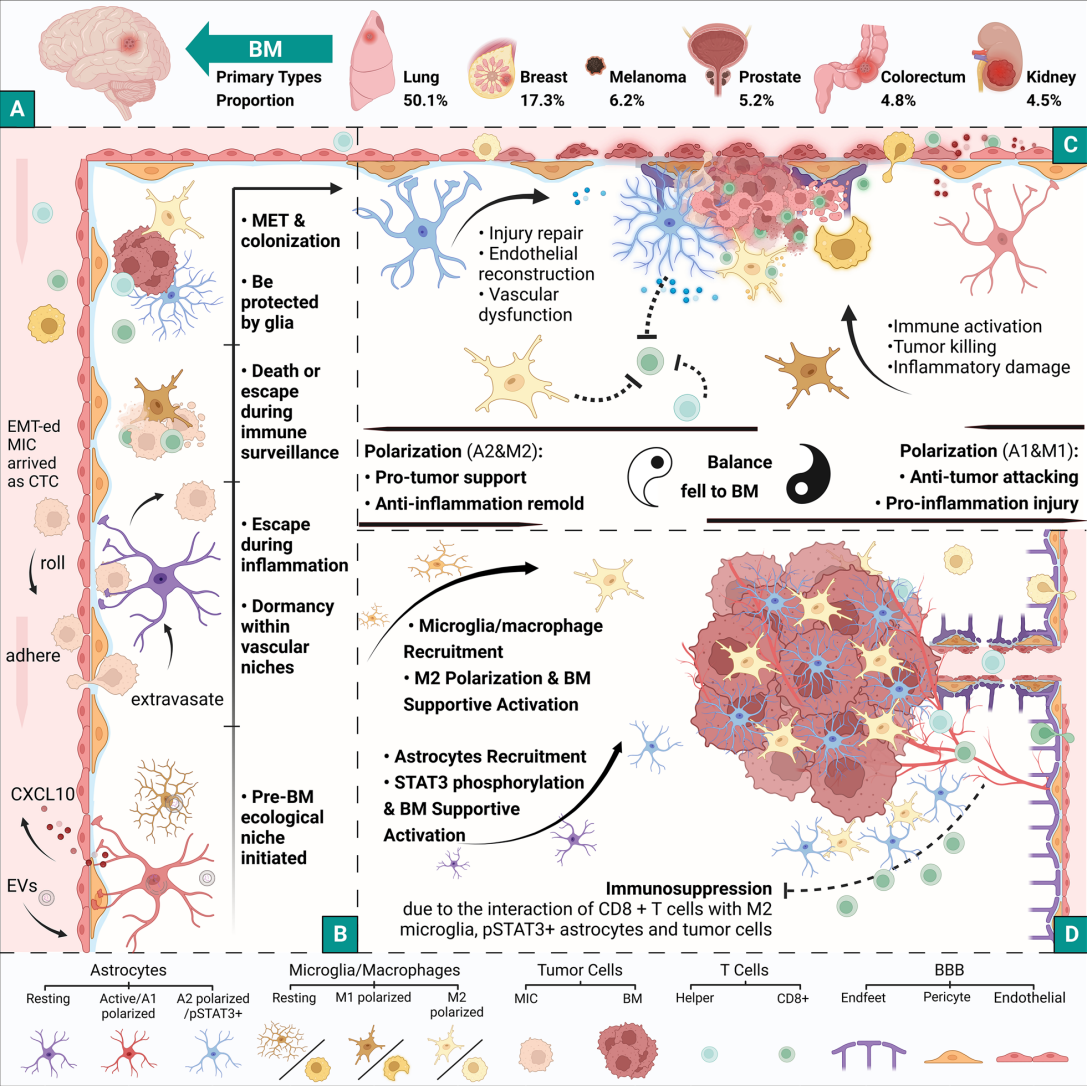
Schematic illustration of the BM program. **A** The most common BMs are from the lung, breast and melanoma, and less from the prostate, colorectum, kidney and other organs. **B** The process of MIC entry into the brain and BM colonization. **C** BM seizes the opportunity for survival in the brain due to the imbalance of the inflammatory response in two types of polarized brain cells. **D** BM fortress with immunosuppressive defenses and support from glial cells is finally formed. Created with BioRender.com. BM, brain metastases; MIC, metastases-initiating cell; CTC, circulating tumor cell; EMT, epithelial–mesenchymal transition; MET, mesenchymal–epithelial transition; EVs, extracellular vesicles; CXCL10, C-X-C motif chemokine 10

An estimated 8–10% of cancer sufferers develop brain metastases (BM), equating to nearly 200,000 novel cases per year in the United States, as believed by the ASCO-SNO-ASTRO Guideline [1]. Johns Hopkins University conducted a recent study indicating the top 3 primary sources of BM to be lung (50.1%), breast (17.3%) and melanoma (6.2%) cancers. Other significant contributors include malignancies from prostate (5.2%), colorectum (4.8%) and kidney (4.5%) (Fig. 1A). Additionally, the BM from urinary system together represents about 12%, and the BM rate for esophagus cancer surpassed only by lung cancer according to reported data [2]. More infrequent primary BM types include hepatocellular and thyroid carcinomas, accounting for 0.2–2.2% and 1–2%, respectively [3, 4]. An analysis conducted by Brigham and Women’s Hospital, using the Surveillance, Epidemiology and End Result (SEER) database from the USA, revealed median survival times for BM sourced from lung, breast and melanoma cancers to be between 4.0 and 6.0, 10.0 and 6.0 months, respectively. The median survival (quartiles) of overall patients with BM is noted as 5.0 (2.0, 12.0) months [5].

Domestic and World Health Organization (WHO) surveys consistently rank lung cancer as the leading cause of malignant tumor-related deaths in China [6, 7]. Chinese clinical practice guidelines data suggest 20–65% of lung cancer patients will experience BM during disease progression with lung cancer as the most common type of BM [8]. Different histological types of lung cancer present varying BM risks. For instance, the SEER survey reports that 9% of non-small cell lung cancer (NSCLC) patients have BM, with incidences for large cell, adeno and squamous cell carcinoma noted as 12%, 11% and 6%, respectively. Additionally, 18% of small cell lung cancer (SCLC) patients receive a BM diagnosis [9]. It is generally believed that approximately 10–20% of newly diagnosed NSCLC patients present with BM [10]. Two-thirds of NSCLC with BM develop multiple intracranial lesions [11]. The BM incidence of SCLC patients at the first visit is also 10%, but this proportion escalates to 40% during disease progression [8, 12].

Globally, breast cancer, with both incidence and mortality rates ranked highest among women [7], also contributes to a significant BM incidence percentage. As per the Chinese consensus, around 15% of patients with advanced breast cancer will develop central nervous system (CNS) metastases [13]. Advanced breast cancer patients with HER2-positive or triple-negative molecular subtype exhibit a higher incidence of BM [14].

Despite the relatively lower incidence of melanoma, its high malignancy rate, poor prognosis and predisposition for metastasis have made it one of the top three common sources of BM [7, 15]. More than 40% of stage IV melanoma patients will develop BM [16, 17]. BM directly causes 60–70% of fatalities in melanoma patients, as stated by German management recommendations for melanoma BM (MBM) [17].

### BM formation

BM formation is a convoluted process (Fig. 1B): (i) metastases-initiating cells (MICs), which have achieved epithelial–mesenchymal transition (EMT) detach from the primary tumor and permeate the bloodstream, subsequently becoming circulating tumor cells (CTCs) that reach the intracranial region following circulation; (ii) after adhering to the vascular endothelium, the MIC crosses the BBB, akin to leukocytes, and then enters a dormant phase again in the vascular niche formed by astrocytes and the vessel wall; (iii) the MIC develops potential to be metastases after avoiding astrocyte limitation, and evading surveillance and elimination by immune cells, such as microglial/macrophages; (iv) mesenchymal–epithelial transition (MET), immune evasion, migration, colonization, growth and proliferation of MIC and micro/macrometastases are accomplished via interactions with brain cells [18–21]. This is fundamentally the classic “seed-soil” theory, wherein the metastatic cell “seeds” with specific gene mutations adapt to and remodel the brain microenvironment “soil”. Concurrently, the “soil” screens the “seeds”, allowing the tumor to survive [21, 22].

Understanding BM requires a perspective encompassing immunity and inflammation. T cells are involved in infiltrating BM, with higher CD8 + and CD45RO + T cell densities associated with improved prognosis, whereas immunosuppressive CD4 + CD25 + FOXP3 + T helper cells demonstrate the opposite effect. However, compared to primary extracranial tumors, T cells in BM show diminished infiltration, clonal expansion and diversity [23, 24].

Although it is still disputed whether astrocytes with structural support and homeostasis maintenance functionalities aid or hinder BM growth during the initial stage, substantial evidence points toward reactive astrocytes promoting metastases during tumor interaction [23, 25]. Astrocytes help metastatic cells achieve immune escape: signal transducer and activator of transcription (STAT3)-positive astrocytes amplify the number of supportive CD74 + microglial/macrophages in the tumor vicinity and restrict CD8 + T cells from penetrating the tumor microenvironment (TME) as a barrier (Fig. 1C) [26].

Microglia, members of the macrophage system, act in immune surveillance in the CNS [27]. However, in response to microenvironmental changes, microglia exhibit a spectrum of alteration corresponding to a dual role, with two extremes that can be understood as polarized phenotypes: namely the proinflammatory, antitumor (M1) polarization, which elevates inflammatory cytokine levels and strengthens T cell-mediated antitumor response; and the contrasting anti-inflammatory, protumor (M2) polarization that stimulates angiogenesis and tumor growth (Fig. 1C) [23, 28, 29]. Andreou et al. [30] illustrated that targeting the anti-inflammatory phenotype in continuously infiltrating microglia/macrophages in BCBM significantly decreased tumor growth. Qiao et al. [31] demonstrated that activated microglia/macrophages expressing matrix metalloproteinase (MMP) 3 accumulate in MBM and play a role in promoting the tumor.

In the context of neuroinflammatory reaction, astrocytes and microglia quickly mobilize to counter invading agents and repair tissue damage by identifying damage-associated molecular patterns (DAMPs) or pathogen-associated molecular patterns (PAMPs). However, excessive damage load prolongs glia activation, augmenting barrier permeability, foreign immune cells’ recruitment and serving as a pathological source for many CNS disorders [18].

In essence, the BMS microenvironment can be considered a hotbed of unregulated neuroinflammatory responses where alterations in the normal BBB facilitate metastases, and glia cells activated to repair damage become tumor providers instead. During the ongoing inflammation, spent astrocytes and microglia differentiate toward an anti-inflammatory and damage reduction profile, ironically obstructing leukocytes from assail invaders (Fig. 1C&D). This vicious cycle enables rampant BM growth.

### BM therapy

The recent rise in the incidence of BM may be attributed to the effective control of extracranial disease, which prolongs the survival of patients, thereby increasing the likelihood of malignant cells migrating to the brain. Meanwhile, drugs developed for primary lesions may not effectively control intracranial lesions due to factors such as poor blood–brain barrier (BBB) permeability, changes in the normal BBB physiology caused by the tumor, tumor heterogeneity, or the unique intracranial microenvironment [32–34]. It must be noted that the primary contradiction in the inefficacy of previous therapy strategies to control intracranial progression lies in the potency of the drugs reaching the lesion. When in the micro-metastatic phase or early metastases, with the normal structure and function of the BBB, the main aspect of the primary contradiction concerns the BBB permeability of the drug. If single malignant cells or small cancer nests cannot be inhibited, they will ultimately evolve into macro-metastatic lesions, forming new tumor blood vessels and blood-tumor barrier (BTB). At this point, the main factor determining the intracranial efficacy will shift to the drug’s own tumor suppression ability [35, 36]. Additionally, advances in imaging technology, which have enhanced diagnostic efficiency and detection rates, may also contribute the increased incidence [32].

The advent of new-generation targeted drugs brings hope for the control of intracranial progression. In the therapy of NSCLC BM, osimertinib, as a representative third-generation epidermal growth factor receptor (EGFR)—tyrosine kinase inhibitors (TKIs) drug, demonstrated significantly enhanced BBB permeability compared to previous generations and delayed the trend of new CNS metastases in patients without baseline BM [37, 38]. Third-generation anaplastic lymphoma kinase (ALK)-TKIs, including brigatinib, ensartinib and lorlatinib, each achieved an intracranial objective response rate of 70% in clinical trials (NCT02737501, NCT03215693 and NCT03215693) involving patients with ALK fusion gene-positive NSCLC [39–41]. C-ros oncogene 1 receptor tyrosine kinase (ROS1)-TKI representative drug entrectinib is also capable of effectively controlling the risk of CNS progression in ROS1-positive NSCLC patients without baseline BM [42]. BRAF/MEK inhibitor combined strategies, including dabrafenib plus trametinib and encorafenib plus binimetinib, benefit advanced melanoma patients with BRAF gene mutations [43, 44]. Meanwhile, the systematic strategy of combining BRAF/MEK inhibitors with radiotherapy and immunotherapy also brought hope to patients with melanoma MBM [45].

However, targeted therapies still have limitations due to their dependency on patient sensitivity and the variability of persistence to the targeted responses. Besides, the efficacy for patients with baseline BM is inferior compared to those without baseline BM. It is critical to emphasize that the treatment of BM is not a solo effort of any single strategy, and multidisciplinary management should receive adequate attention [46, 47]. Furthermore, it is necessary to explore more deeply the microenvironment of BM tumors. An enhanced understanding of the interactions between BM and brain cells, as well as the intracranial immune response, will aid in our comprehension of the disease and in the discovery of novel, more effective therapy strategies.

Studying BM mechanisms offers insights into therapeutic strategies. Presently, surgical resection and localized radiotherapy, incorporating whole-brain radiotherapy (WBRT) and stereotactic radiotherapy (SRS), constitute the crux of multidisciplinary BM management [46, 48]. Although advancements like laser interstitial thermotherapy (LITT), focused ultrasound (FUS) and advanced imaging technologies offer hope, they struggle with managing multiple intracranial metastases, controlling residual malignant cells at the margin of the surgical cavity and achieving satisfactory survival rates after diagnosis [46, 47, 49, 50]. However, research focused on tumor molecular pathways and the mechanism of cell interaction in the TME has paved the way for novel targeted and immune therapy strategies [46, 47].

In this review, we aim to construct a comprehensive picture of the TME crucial to BM survival. We will then contextualize the progress in immunotherapy predicated on the TME, focusing on immune checkpoint inhibition, bioengineered T cell assistance and tumor–brain cell intervention.

## Microenvironment of brain metastases

### Blood‒brain barrier

The BBB, from the lumen side of the brain capillaries to the side of the parenchyma, is composed of vascular endothelial cells (VECs), pericytes that envelop the endothelial cells and form a basal lamina, and the endfeet of astrocytes. The BBB facilitates the selective exchange of substances between the cerebrovascular and parenchymal compartments, contributing to the isolation of these environments and the maintenance of cerebral homeostasis [36, 51]. Considering its active molecular transport system and peripheral immune cell regulation, the BBB functions as more than a mere physical barrier. For example, claudin, occludin and junction adhesion molecule are transmembrane proteins composing the tight junction (TJ) between the VECs; however, these proteins are not only structural components, but also key factors of the paracellular channel formation, transmembrane transport and even the migration of angiogenic cells to the brain [52]. Consequently, TJs are crucial structures not only to the integrity of the BBB but also to its selective permeability [53]. Moreover, variations in protein expression, affecting permeability and exogenous cell migration, on the BBB among different individuals profoundly influence the invasion of CTCs derived from primary tumors into the brain [18].

Nduom et al. [33] highlighted that, in the case of BM, disruptions to the astrocytic elements of the BBB and subsequent reconfiguration of the astrocyte–endothelial cell relationship occur. Newly formed vascular structures in intracranial tumors present with more convolutions, distinguishing them from both other tumor types and normal vessels. Imbalances in vascular endothelial growth factor (VEGF) expression can foster a hypoxic environment, instigating hypoxia-inducible factor 1α (HIF1α)-mediated transcription programs and thus accelerating tumor progression [36]. It’s noteworthy that BTB of metastases exhibits reduced structural integrity and increased permeability compared to the normal BBB, in addition to inconsistent distributions [18, 36]. The investigation by Godinho-Pereira et al. [54] corroborated that breast cancer metastatic cells enhance both paracellular and transcellular permeability of the BBB, causing significant damage to epithelial integrity and cytoskeletal alterations. They also highlighted the pivotal role of adhesion molecules FAK and β4-integrin in promoting malignant cell migration during the extravasation process.

Reviewing lung cancer BM, Wang et al. [53] summarized the process of CTCs crossing the BBB. Initially, primary tumor cells undergo EMT to invade vessels, subsequently becoming CTCs that reach the brain, particularly regions with slow blood flow such as the gray-white matter junction and vascular boundary area. With the binding of the ligands (on metastatic cells) and adhesion molecules (on endothelial cells), CTCs adhere and exit from the BBB like leukocytes and subsequently undergo mesenchymal-to-epithelial transition (MET) to adapt to the new TME.

Astrocytes, besides their TME modulating role, may directly mediate CTCs passage across the BBB [51]. Astrocytic secretion of MMPs can induce basement membrane type IV collagen degradation, thus enhancing BBB permeability and tumor cell invasiveness [55]. Pathways such as the transforming growth factor (TGF)-β1-mediated exosomal lnc-MMP2-2 found in NSCLC can also increase BBB permeability and encourage brain metastases, further supporting the MMP system’s importance in BBB crossing [56]. CXCL10 chemokine secreted by astrocytes has been shown to elevate the receptor CXCR3 expression in tumor cells, thereby boosting absorption and migration capacity of melanoma BM cells [57]. Meanwhile, the expression of astrocytic sphingosine-1 phosphate receptor 3 (S1P3) in tumor cells could trigger astrocytes to secrete interleukin (IL)-6 and C–C motif chemokine ligand (CCL)-2, promoting endothelial cell adhesion dissolution and thus increasing BBB permeability [58]. Interestingly, an increase in desmin + pericyte coverage might correlate with higher BBB transitability of metastatic cells as well [59].

In conclusion, the structural components of BBB, especially astrocytes, contribute substantially to the formation of BM during the process of interactions with metastatic cell.

### Brain microenvironment: the ecological niche for BM

The brain microenvironment (BME) serves as an intricate and functionally specialized ecosystem constructed by various cell types. This repertoire includes neurons forming the electric signal transmission network, oligodendrocytes constituting the myelin sheath, astrocytes maintaining environmental homeostasis and microglia from the macrophage system. Additionally, pericyte and endothelial cells jointly form the blood–brain barrier (BBB) with astrocyte endfeet while ependymal cells alongside the choroid plexus establish the blood–cerebrospinal fluid barrier. Moreover, immune cells are recruited within this system from the circulatory environment [18, 27].

In metastatic cells, originating from distant primary lesions, those with brain-tropic ability can infiltrate this unique organ. These cells interact with and subsequently restructure the BME to create suitable conditions for their maintenance and proliferation in the brain. Eventually, with growth support and immune suppression, a community of tumor environments gradually forms, completing the hypothetical process of “seed” colonization in the “soil” [60–62]. Astrocytes, microglia/macrophages and recruited T cells are the BME components of primary concern due to their significant interaction with metastases.

The formation of a specialized ecological niche may initiate even before CTCs penetrate the BBB. This early development may finally morph into an invasive structure, bolstered by BME components, under the influence of selective pressure from heterogeneity [63].

It has been proven that astrocyte and microglia activation is strongly linked to early BM growth [64]. Astrocytes secrete chemokines that attract helper T cells, tumor-associated macrophages and microglia to the tumor site, inhibiting the cytotoxic effects of the recruited CD8 + T cells, thus indirectly supporting BM [65]. Furthermore, glial cells enhance tumor cells’ immune evasion directly. For instance, exosomal microRNA secreted by astrocytes induces PTEN downregulation in tumors, fostering CCL2 secretion and supportive myelocyte recruitment [66]. The crosstalk between the BME and BM cells involves various secretory products such as serum protein E1, interleukin-8 and secretory phosphoprotein 1 that correlate with tumor invasiveness [67].

Moreover, the high energy consumption processes in neurons dictate a competitive environment for oxygen and nutrients between neurons and BM cells. The typical example of adapting to this milieu is that breast cancer cells colonizing in the brain manifest a neuron-like γ-aminobutyric acid energy phenotype [68]. An upregulation of neurotrophin receptor expression stimulated by endogenous neuron growth factors further supports cancer cell proliferation [69]. Over time, metastatic cells possibly reconfigure the BME to favor their proliferation. Astrocytes are critical supporting cells in the BME and have the function of damage repair. Malignant cells can induce the differentiation of neural stem cells into astrocytes via paracrine BMP-2 signals in situations where they escape from the competition with these indigenous cells, thereby facilitating metastases survival during the long term of colonization [70].

Both within and outside the tumor, increased Ki67 expression related to microvascular proliferation and inhibited connexin claudin-5 expression related to vascular permeability in endothelial cells engender a mutually beneficial coexistence between the tumor and the vessels within the BME [71].

Collectively, while BME heterogeneity presents an obstacle hindering malignant cell metastases, it potentially provides an ecological niche for BM. The interaction between tumor cells, inherent cerebral cells and recruited cells facilitates BME remodeling, leading to the emergence of a TME.

### Tumor microenvironment

Brain metastases are associated with a distinctive TME. The TME of BM may differ significantly from not only the primary tumors but also CNS primary tumors. Karimi et al. [71] discovered that lung, breast and melanoma BM share a similar core environment irrespective of the primary type, but the marginal environment is similar to glioblastoma with frequent cell–cell interactions. Klemm et al. [72] also demonstrated that the BM model exhibited significant aggregation of lymphocytes and neutrophils different from glioma; however, the aggregation of CD4 + and CD8 + T cells in melanoma BM was higher and neutrophil infiltration was stronger in breast cancer BM.

The TME in BM also exerts control over the immune response, influencing detrimental inflammation and restricting the infiltration of cytotoxic T cells. It also aids in immune evasion and facilitates immune downregulation by microglia/macrophages, thereby promoting tumor survival and drug resistance [73]. In BM, particularly in the basal-like/triple-negative breast cancer (BLBC/TNBC) subtype, human leukocyte antigen-A (HLA-A) DNA methylation or focal deletion hinders the activation of the CD8 + T cell immune response during the recognition stage [74]. On the other hand, the downregulation of CX3CR1 in central nervous system (CNS) myeloid cells within the TME results in increased secretion of the chemokine CXCL10, which promotes the recruitment of immune checkpoint (VISTA and PD-L1) positive myeloid cells and inhibits T cell immune response activity during the negative regulation stage [75]. In addition, Rubio-Perez et al. [76] observed that the phenotypes of cytotoxic lymphocytes in the cerebrospinal fluid (CSF) were consistent with those present in the TME, thereby expanding our understanding of the TME and supporting the potential for noninvasive monitoring of TME changes using CSF.

While BM shares similar pathways of formation and colonization, it is crucial to acknowledge the significance of multiple sources, necessitating comprehensive research on the diverse primary tumor types and their corresponding TMEs [63, 71].

Studies on NSCLC have revealed that BM exhibits poorer immune-related functions compared to primary tumors. This includes lower proportions of tumor-infiltrating lymphocytes (TILs), reduced expression of programmed death ligand 1 (PD-L1), decreased signals related to interferon γ and upregulation of anti-inflammatory markers TOLLIP and HLA-G. These findings indicate the presence of a more immunosuppressive microenvironment within the metastatic lesions [77, 78]. It is noteworthy that, in comparison to EGFR wild-type lung adenocarcinoma, immune-related pathways were upregulated in EGFR-mutated BM, even though no significant differences were observed in the primary tumors with EGFR mutations [78]. Furthermore, TP53-mutant lung BM demonstrated higher levels of CD8 + T cell activation and infiltration. However, there was also a significant accumulation of immunosuppressive tumor-associated myeloid cells (CD45 + CD11B +), along with a downregulation of proinflammatory factors [79]. These observations suggest a close association between the formation of the TME immune landscape and the tumor’s inherent characteristics driven by specific gene mutations.

The TME of intracranial breast cancer lesions exhibits significant immunosuppressive characteristics, characterized by the presence of FOXP3 + regulatory T cells (Tregs), LAMP3 + dendritic cells, CCL18 + M2 macrophages, RGS5 + tumor-related fibroblasts and LGALS1 + microglia [80]. This TME actively inhibits the activation of CD8 + T cells and facilitates the recruitment of other immunosuppressive cell populations. Griguolo et al. [81] further demonstrated that higher concentrations of anti-inflammatory (M2) microglia/macrophages in HER2- brain metastases predicted worse outcomes. Additionally, a closer spatial association between immune cells expressing programmed death-1 (PD-1) and PD-L1 in HER2 + brain metastases was linked to poorer survival.

Intriguingly, immune checkpoint pairs such as LAG3-LGALS3 and TIGIT-NECTIN2 play a dominant role in immune evasion in BCBM, rather than the well-known PD1-PDL1/PDL2 interaction [80]. Sirkisoon et al. [82] discovered that BCBM, particularly those enriched in HER2-positive and TNBC, exhibit high expression of the truncated glioma-associated oncogene homolog 1 (TGLI1), a tumor-promoting transcription factor. TGLI1 strongly stimulates astrocytes, leading to modifications in the microenvironment and mediating the formation of brain metastases. Interestingly, they elucidated the interaction between HER2-positive BCBM cells and astrocytes. Astrocyte-secreted brain-derived neurotrophic factor binds nonspecifically to the tumor-expressed TrkB receptor, facilitating heterodimerization between TrkB and HER2. This, in turn, transmits signals for colonization and proliferation via the PI3K pathway [83]. However, further investigation of the TME is warranted to elucidate the potential interconnections between these mechanisms and the underlying reasons for the high burden of brain metastases in TNBC. Additionally, Álvarez-Prado et al. [79] identified a subset of kataegic BCBM with a notably high mutation burden through genomic analysis. This subset exhibited a significant increase in the proportion of CD103 + CD8 + and CD4 + T cells, accompanied by a decrease in neutrophil infiltration, suggesting a prominent proinflammatory phenotype.

Among the common types of BM, melanoma has the highest level of metastases-related inflammation, followed by lung, and breast cancer has the lowest. This means that melanoma has a relatively higher accumulation of microglia/macrophages and peripheral infiltration of CD8 + T cells [71]. However, there is a large proportion of dysfunctional TOX + CD8 + T cells in MBM with significant chromosomal instability [84]. BRAF mutations occur in half of melanomas, accounting for 75.4% and 17.2% of V600E and V600K, respectively [85] PI3K-AKT signaling may also be a prerequisite for MBM, which can be stimulated by various astrocyte secretions. However, the neurotrophic factor PTEN is downregulated in the TME. Moreover, IL23 secreted by astrocytes activates the JAK2-STAT3 pathway, and CCL17 guides CCR4 + melanoma cells, both of which lead to the expression of tumor-promoting substances [61]. Pozzi et al. [65] found that astrocytes, under the influence of the TME, upregulate the secretion of monocyte chemoattractant protein-1 (MCP-1), which induces tumor cells to overexpress the receptor CCR2 and ultimately affects multiple adverse effects, such as immunosuppressive cell recruitment, microglial/macrophage anti-inflammatory/tumor-promoting polarization and CD8 + T cell suppression.

Apart from the critical influence of astrocytes on the TME, researchers have discovered the special roles of other cells as walls. Alvarez-Breckenridge et al. [86] identified three different states of neutrophils in the TME: high calprotectin, interferon (IFN) response and high IL8. Overexpression of IL8 may be associated with EMT in metastatic cells. Smalley et al. [87] study on MBM highlighted a specific type of dendritic cell that improves the survival rate and treatment response. It does so by promoting the T cell response and increasing the expression of major histocompatibility complex (MHC) in tumor cells. Furthermore, metastatic malignant cells even exhibit significant neuronal-like differentiation tendencies. Biermann et al. [84] illuminated the sustained upregulation of multiple neuronal differentiation genes in MBM, particularly the high enrichment of NCAM1. It may be the “cunning” aspect of the BM actively adapting to the survival environment under the BME.

### Astrocyte

Astrocytes, acting as fundamental constituents of the BBB and the BME, perform essential structural and supportive functions, significantly influencing metastatic cell migration from extracranial sites [27, 51]. For instance, TGLI1, identified as a tumorigenic transcription factor, fosters the central genes of BM stem cells, notably SOX2 and OCT4. These genes correspond significantly with diminishing brain-free survival concurrent with TGLI1 + stem cells demonstrating intensive cell–cell interactions with astrocytes [82]. Breaking the spatial restriction-mediated dormancy of astrocytes by oligomeric metastatic cells may be a speed-limiting step in BM (Fig. 1B) [88]. Dystroglycan (DAG) on the membrane of dissected tumor cells (DTCs), which are in the niche enclosed by the endfeet of astrocytes and the cerebral vascular wall, was stimulated by astrocyte-derived laminin-211. This relationship places them beyond the nucleus combined with yes-associated protein (YAP), obstructing the BM-promoting growth program [88, 89]. Alas, as the cornerstone of this resting mechanism, laminin shows high vulnerability to inflammatory damage [90].

Indeed, before the encroachment of the brain commences, astrocytes begin serving a supportive role in the BM ecological niche (Fig. 1B). General Lahav et al. [91] detected copious RNA levels of several recognized inflammatory activators in melanoma-secreted extracellular vesicles (EVs), including Hmgb1, Tslp and Irf1. These tumor exosomes indeed incited the proinflammatory signals of primary astrocytes. Intense activation of astrocytes is also TGLI1 + breast cancer related ascribed to EVs [82]. The miRNA-1290 transported by EVs, secreted from primary lesions into the brain, inhibits the ciliary neurotropic factor (CNTF) transcription inhibitor forkhead box A2 (FOXA2), leading to the CNTF upregulation—an established brain survival factor. This significantly activates astrocytes, promoting BM growth, while confirming higher expression levels of the CNTF α receptor (CNTFR-α) in BM compared to primary tumors [92].

Astrocytes play an essential role in facilitating tumor metastases, proliferation and expansion. Upon upregulation of CXCL10, an inflammatory chemokine derived from astrocytes related to metastases, encephalophilic melanoma cells can correspondingly elevate the receptor CXCR3. This alteration manipulates inflammatory recruitment signals, aiding astrocytic metastases and brain colonization (Fig. 1B) [57]. Pervasively, metastatic tumor stem cells infiltrating the brain augment the release of IL-1β, enhancing the expression of JAG1 in astrocytes. This significant activation elicits notch1 signaling during the astrocytes-stem cells interaction, leading to the upregulation of the transcription factor HES5. Subsequently, the tumor stem cell population undergoes self-renewal [93].

Furthermore, the proliferation of BM requires the activation of peroxisome proliferator-activated receptor gamma (PPARγ) situated within the tumor nucleus, forming heterodimers with RXR to trigger related genes [94]. The primary stimulus for PPARγ signaling is the brain’s elevated lipid environment due to arachidonic acid and mead acid released by astrocytes. Besides, Gong et al. [95] addressed that astrocytes are stimulated by IL-1β and TNF-α, derived from TNBC, resulting in an upsurge of TGF-β2 expression. Consequent activation of the TGF-β receptor-1/phospho-SMAD3 pathway results in the formation of the nuclear gene transcription regulatory factor SMAD2,3/SMAD4 complex. This subsequently upregulates the expression of secreted glycoprotein ANGPTL4, fostering metastatic tumor growth in the brain.

STAT signaling plays a vital role in the interaction between astrocytes and BM. Various secreted factors from BM cells stimulate astrocyte STAT3 signaling, leading to alterations in the expression of immune regulatory factors. This, in turn, upregulates the immune checkpoint PD-L1, inhibiting cytotoxic CD8 + T cells, while activating the MIF-CD74-midkine axis to recruit tumor-supporting microglia. These processes significantly impact the survival and growth of BM [26, 96]. Furthermore, astrocyte-mediated STAT3 response in BM can also result in cerebrovascular dysfunction, leading to neurological impairment [97].

Aside from STAT3, STAT1 is another noteworthy focus for BM researchers. Gap junction-mediated interaction between astrocytes and BM cells induces the upregulation of IFNα and TNF via the cGAMP-STING signaling pathway, activating STAT1 and NF-κB p65, respectively. This activation promotes tumor growth and resistance [25]. Additionally, the further indication of astrocyte-mediated type I interferon release suggests that it also exerts a pro-BM effect by increasing the recruitment of immunosuppressive myeloid cells [98]. However, Turnquist et al. [99] have highlighted that inflammatory signals, particularly lipopolysaccharides, can upregulate STAT1 in tumors, targeting the pro-transcription of the tumor suppressor factor ASPP2. ASPP2 has the potential to induce tumor apoptosis directly or indirectly promote P53 action. It may be that the protumor and antitumor effects of STAT1 signaling represent opposite ends of a feedback cycle, or there may exist an undiscovered underlying transcriptional mechanism that targets ASPP2. Undoubtedly, research on STAT signaling in BM holds considerable potential with practical applications.

**Fig. 2:**
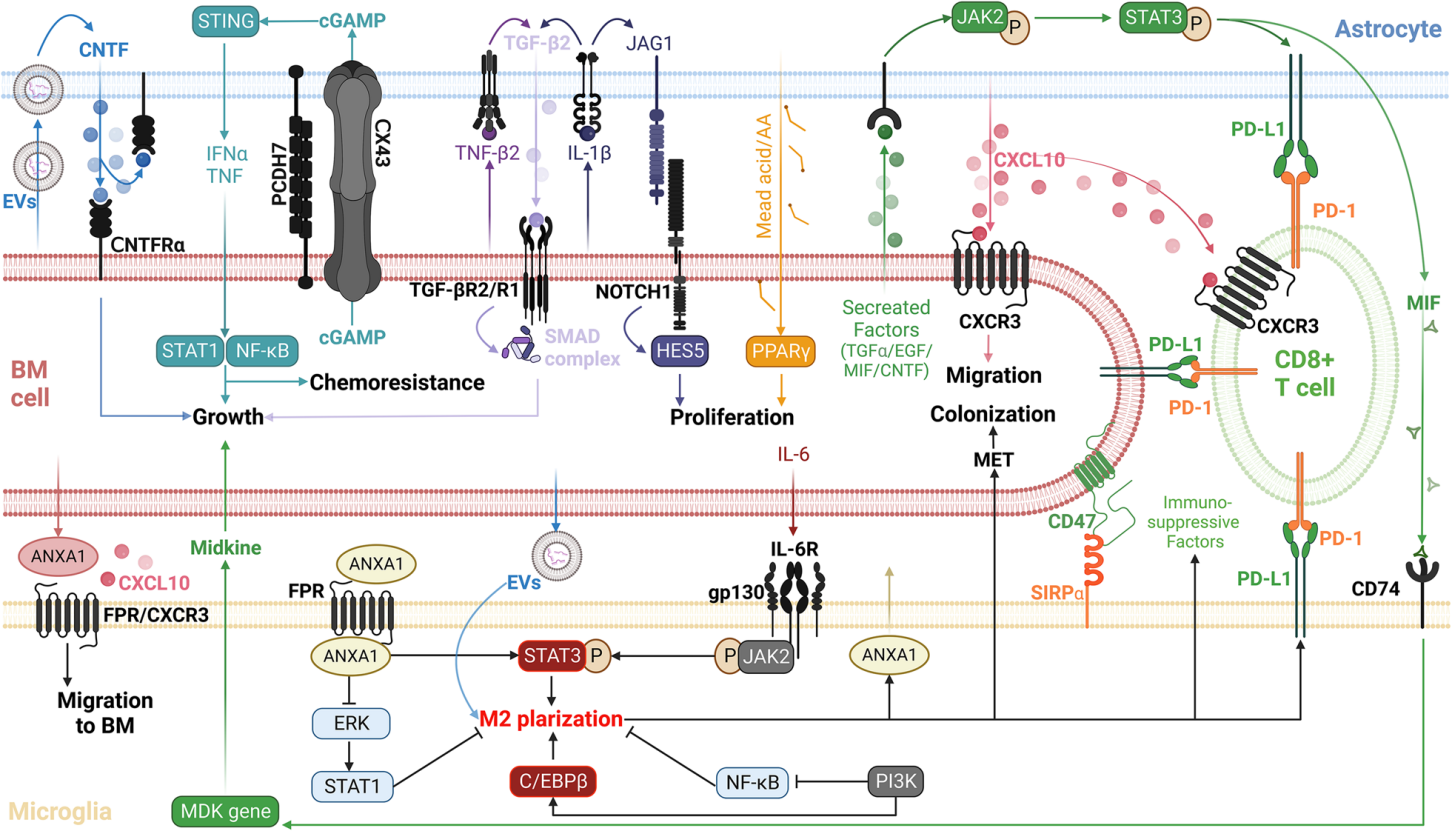
The mechanism of the interaction between BM, glia and cytotoxic T cells. Tumor-derived EVs could remotely activate astrocytes and microglia, remolding the premetastatic ecological niche. Gap junctions are the critical pathway for the interaction between astrocytes and BM, forming a cycle in which the two promote activation or survival. TGF-β2 secreted by astrocytes, which are stimulated by inflammatory factors released from the tumor, also promotes BM growth. The upregulation of JAG1-NOTCH1 signaling caused by BM and the high lipid environment originally formed by astrocytes itself both increase malignant proliferation. Moreover, a variety of BM secretion factors upregulate the phosphorylation level of STAT3 in astrocytes, not only upregulating the expression of PD-L1 but also MIF, which combines with CD74 of tumor-supporting microglia to promote BM growth. Signal activation, including STAT3 and PI3K, will cause the anti-inflammatory and tumor-promoting M2 polarization of microglia, while PI3K or microglia-derived ANXA1 (tumor-derived ANXA1 mainly influences migration) will inhibit the anti-M2 signal NF-κB or STAT1. CXCL10 is an important migration guiding marker in the TME. Although it also leads to the recruitment of CD8 + T cells, they will be fettered by multiple immune checkpoints, resulting in immune effectiveness being masked. Created with BioRender.com. CNTF, ciliary neurotropic factor; cGAMP, cyclic GMP-AMP; CX43, connexin 43; PCDH, protocadherin; STING, stimulator of interferon genes; INFα, interferon alpha; TNF, tumor necrosis factor; STAT, signal transducer and activator of transcription; NF-κB, nuclear factor kappa-B; TGF, transforming growth factor; IL, interleukin; JAG1, jagged1; HES, hes family bHLH transcription factor; AA, arachidonic acid; PPARγ, peroxisome proliferator-activated receptor gamma; EGF, epidermal growth factor; JAK2, Janus kinase 2; MIF, macrophage migration inhibitory factor; SIRPα, signal regulatory protein alpha; gp130, glycoprotein 130; PI3K, phosphoinositide 3-kinase; C/EBPβ, C/CAAT enhancer binding protein beta; ANXA1, annexin-A1; FPR, formyl peptide receptor; ERK, extracellular signal-regulated kinase

Together, astrocytes and tumor cells exhibit significant interaction during BM progression. Prior to metastasis, noncoding RNAs carried by EVs derived from primary cancers initiate the remodeling of the brain’s ecological niche by inducing astrocytic changes. However, astrocytes, which mediate the dormancy of disseminated tumor cells DTCs, face challenges due to local inflammation that can damage the fundamental substance laminin. Additionally, a range of signaling axes exists between astrocytes and BM cells, including the CXCL10-CXCR3, JAG1-Notch-HES5, fatty acid-PPARγ, TGFβ2-SMAD-ANGPTL4, STAT3 signaling pathways and the gap junction-mediated cGAMP-STING-IFNα/TNF-STAT1/P65 axis, all of which critically influence BM metastasis, proliferation, growth and immune evasion (Fig. 2).

### Microglia

Microglia are the primary innate myeloid immune cells present in the brain [27]. Research has identified significant microglia activation and aggregation at the boundaries of BM lesions, coupled with infiltration into the tumor [18, 100]. A strong correlation exists between the area of myeloid cell markers and tumor volume, while proinflammatory and anti-inflammatory markers are maximally expressed in metastatic lesion cores [30]. Additionally, the interaction between microglia and BM cells is reciprocal. Microglia stimulate BM cell migration across the endothelium, modulate the expression of specific genes that promote proliferation and impact the levels of ERK (an inhibitor of tumor growth) and STAT3 messenger phosphorylation (a promoter of tumor proliferation) [101, 102]. Conversely, BM influence the proliferation, M2 phenotype polarization and tumor-oriented migration of microglia while inhibiting the inflammatory response [101, 103]. Further, MMPs in the TME dissolve the matrix to enhance migration, given the marked upregulation of BM and microglia-derived MMP2 and MMP3 due to tumor–glia interactions [31, 101].

STAT3 signaling bears significant impact on the microglia transformation from the proinflammatory/antitumor M1 phenotype to the anti-inflammatory/tumor-promoting M2 phenotype. This constitutes a key signal mediating BM’s immunosuppressive microenvironment [18, 104]. The inflammatory factor IL-6 secreted by BM cells is an important upstream signal that activates the IL6R-JAK2-STAT3 signaling pathway and the potential target gene ARG1 of STAT3, thus inducing M2 polarization of microglia, which in turn induces MET and brain colonization of metastatic cells [103]. Interestingly, the exogenous substance nicotine can also promote the M2 polarization of microglia through the α7nAchR-JAK-STAT3 pathway. This polarization leads to the secretion of the chemokine ligand CCL20 and insulin-like growth factors, which in turn promote the formation and growth of tumor colonies. Additionally, nicotine facilitates immune evasion through the SIRP-CD47 signaling pathway. In particular, the expression of CD47 is upregulated in lung cancer BM patients with a smoking history [105].

Another important upstream signal of STAT3 is annexin-A1 (ANXA1), which can be divided into two types with three sources. These sources are exogenous tumor secretion, exogenous microglia secretion and endogenous microglia. Exogenous ANXA1 activates STAT3 through the formyl peptide receptor (FPR) 1 or 2 pathway. Tumor-secreted ANXA1 drives microglial migration, while microglia-secreted ANXA1 promotes microglial activation and regulates the expression of inflammatory factors [102]. On the other hand, the binding of endogenous ANXA1 enhances STAT3 activation and inhibits the ERK1/2-STAT1 pathway. This inhibition antagonizes STAT3 and promotes the expression of inflammatory genes [102].

A significant downregulation or deletion of the lncRNA XIST in breast cancer BM increases the secretion of microRNA-503. This induces STAT3 phosphorylation in microglia and reduces NFκB phosphorylation, thus facilitating the M1/M2 phenotype reprogramming and elevating the expression of immune checkpoint PD-L1. Moreover, XIST deficiency triggers an epithelial-to-mesenchymal transition and potentiates the MSN-c-Met pathway, thereby enhancing tumor cell stemness [106].

Several noteworthy mechanisms facilitate microglia-mediated immunosuppression. One such mechanism involves the PI3K signaling pathway, primarily active in not only tumor cells but also the BME. It serves as an essential regulator of tumor-associated microglia/macrophages within the BM [107]. This pathway inhibits nuclear factor kappa-B (NFκB p65) to consequently suppress the expression of inflammatory factors. Concurrently, it activates the C/CAAT enhancer binding protein beta (C/EBPβ) thereby promoting the expression of immunosuppressive factors. This dual action initiates the immunosuppressive transcription program, which inhibits CD8 + T cell activation and recruitment, facilitating tumor survival [108]. In addition to the PI3K pathway, microglia/macrophages in the BM could also upregulate the expression of immune checkpoints VISTA and PD-L1, leading to the inhibition of the T cell immune response. Moreover, the potential antitumor benefits mediated by T cell recruitment through the chemokine CXCL10, released from the TME, are negated by the build-up of T cell inhibitory myeloid cells [75].

In an intriguing study by She et al. [109], it was discerned that BM of lung cancer correlated with a diminished expression of interferon-induced transmembrane protein 1 (IFITM1). The high expression of IFITM1 in early BM cells potentiated the release of microglia-activating complement 3 and amplified the expression and membrane localization of MHC I. This phenomenon, in turn, enabled microglia and CD8 + T cells to collaboratively eliminate cancer cells via mechanisms such as interferon γ, cell phagocytosis and T cell-mediated killing. In fact, the presence of IFITM also hints at the potential of interferon responses in BM immunity [110]. Although various pathogen recognition receptors, including the Toll-like receptor (TLR) family or other nucleic acid ligand-sensing receptors, can mediate the release of type I interferon and cancer therapy [111, 112], research on BM has focused on the cGAS-STING pathway in astrocytes as detailed above, which has been shown to protumor effects [25, 98]. The expectation that clarifying or exploiting the relationship between BME cells, interferon responses and BM will open up greater possibilities for immunotherapy.

Not only astrocytes but also microglia are subject to the distant influence of cancer-derived EVs (Fig. 1B). Xu et al. [113] found that LINC00482 released by EVs of NSCLC binds competitively to microRNA-142-3p in microglia. This interaction augments the expression of Transforming Growth Factor Beta 1 (TGF-β1), thereby inciting polarization toward the M2 phenotype in microglia. This microglial alteration not only induces the production of the chemokine CCL16, thereby promoting angiogenesis, but also upregulates the expression of PD-L1 to mediate immunosuppression.

Together, M2 microglia mediate the immunosuppressive TME and support BM colonization and survival. During this process, various upstream signals, such as IL-6, nicotine, ANXA1 and XIST deficiency, play a major role in stimulating STAT3. Moreover, the PI3K-AKT-mTOR-NFκB/C/EBPβ pathway, CXCL10 recruitment signal, IFITM1 deficiency and LINC00482/miR-142-3p/TGF-β1 axis also propelled BM (Fig. 2).

## Immunotherapy targeting the TME

### Immune checkpoint inhibitors

Immune checkpoint inhibitors (ICIs) are at the forefront of BM immunology research, with numerous investigations evaluating their efficacy and characterization in combination with standard treatment protocols [114, 115]. There are some ICIs that are currently receiving the most attention in BM immunotherapy: anti-PD-1 antibodies, such as pembrolizumab and nivolumab; anti-cytotoxic T lymphocyte associate protein-4 (CTLA-4) antibodies, such as ipilimumab and tremelimumab; and anti-PD-L1 antibody, atezolizumab.

When PD-1 on the surface of T cells binds with PD-L1 and PD-L2 expressed on antigen-presenting cells (APCs) or cancer cells, it inhibits the downstream signal transduction of the T cell receptor (TCR). This negative costimulatory signal mediated by PD-1 and its ligands limits the activation of T cells or even exhausts them, thus suppressing the antitumor immune response. In contrast, the immunosuppressive effect of CTLA-4 is based on competitive inhibition of costimulatory signals. The activation of T cells requires not only the stimulatory signal received by the TCR but also the interaction of CD28 on the T cell surface with B7 family molecules (B7-1/CD80 and B7-1/CD86) expressed on APCs to deliver a costimulatory signal. CTLA-4, with higher affinity, competitively binds to B7, mediating maintenance of T cells in a resting state. Additionally, CTLA-4 itself transmits inhibitory signals into the T cell as well. ICIs are part of a feedback mechanism that ensures the moderation of the immune response. However, in the TME, PD-1 and its ligands, along with CTLA-4, are characterized by overexpression, creating immunosuppressive conditions conducive to the survival of malignant cells. The use of antibodies to block the immunosuppressive effects caused by ICIs holds promise as a mature strategy in antitumor immunotherapy [116–118].

In a compelling case study, an elderly Chinese male patient diagnosed with NSCLC, who suffered from multiple instances of BM and brain edema, was reported. Post-administration of Pembrolizumab monotherapy, the patient exhibited complete disappearance of intracranial symptoms, along with a partial response (PR) for chest lesions, which persisted for a significant 15-month period [119]. Although this extraordinary efficacy may represent a unique instance, it undeniably indicates that the therapeutic application of ICIs is progressively establishing in the treatment of BM.

ICIs are deemed more appropriate for patients presenting asymptomatic, small-sized intracranial lesions. It could defer the immediate need for a direct intervention, thereby potentially reducing or even altogether avoiding the risk associated with damage and recovery from surgical excision and/or radiosurgery [48]. Nevertheless, for patients with symptomatic, larger intracranial lesions or for those demonstrating tumor progression in the course of systemic treatment, direct intervention (surgery and/or radiosurgery) should be prioritized in the strategic development of ICI combination therapy; a multidisciplinary approach would yield a more pronounced effect [48]. Additionally, cancer variants tend to manifest varying extents of inflammation surrounding metastatic tumors. This partial variability is attributed to significant inconsistencies in the expression and interplay of immune checkpoints within different tumor immune microenvironments [73]. As a result, personalized therapy is strongly recommended for different types of brain metastatic tumors.

#### Application: general perception based on retrospective study

##### MBM

Lymphocyte infiltration in MBM is prominently dominated by CD8 + T cells, lending these tumors significant sensitivity to ICI therapy [73]. This sensitivity is noteworthy despite the progression to brain metastases in half of the reported cases [48, 115]. A growing number of studies reveal that the combination of SRS and immunotherapy, particularly with ipilimumab and nivolumab, is becoming a conventional treatment strategy for asymptomatic MBM [120]. Further, therapies using BRAF/MEK cell signal transduction pathway inhibitors and PD-1/CTLA-4 immune checkpoint inhibitors supplement and in certain instances actively replace, surgery and radiotherapy (RT) [121].

The real-world study results published by Hilbers et al. [122] demonstrate a distinguishing and sustainable benefit of ipilimumab/nivolumab combination therapy for MBM patients. Interestingly, patients with BRAF gene mutations showed superior efficacy with the combination of ICIs over targeted therapy. Although we must acknowledge potential biases in the reported study, wherein patients with poor prognostic characteristics might prefer targeted therapy, the results continue to be incredibly vital in fostering the use of combined ICI therapy as the primary treatment choice for MBM.

##### NSCLC BM

The prevalence of BM in patients with NSCLC is significant, and it prompts the investigation of ICIs potential applications in this disease context. NSCLC BM typically presents a “cold” phenotype characterized by biological heterogeneity, low PD-L1 expression and minimal lymphocytic infiltration. The TME modulates the dynamic interactions and responses of helper lymphocytes or cytotoxic cells with glial cells in the CNS, specifically, astrocytes marked by phosphorylated STAT3. This intricate network results in malignant cells evading the immune system, which complicates the development of ICI therapies [123].

Nevertheless, ICIs have demonstrated beneficial impacts, primarily by controlling long-term progression of intracranial lesions in advanced NSCLC patients. These beneficial impacts seem to be more pronounced in patients with a PD-L1 tumor proportion score of ≥ 1, along with a low intracranial burden and absence of driver gene mutations [124, 125]. A meta-analysis led by Teixeira et al. [126] established no significant statistical difference between the intracranial response rates of ICI and radiotherapy co-administration and ICI monotherapy for NSCLC BM, hence supporting the notion that immunotherapy promotes favorable intracranial responses.

Despite the optimism surrounding ICI monotherapy’s impact on intracranial active lesions in selected NSCLC patients, one should cautiously consider the potential selection bias introduced by retrospective studies. The future direction entails a comprehensive understanding of the NSCLC BM tumor/immune microenvironment to guide therapeutic strategies and larger-scale, prospective clinical trials. A review of key findings from prospective studies on this topic follows in the next section.

##### Renal cell carcinoma (RCC) BM

The therapeutic approach for RCC BM is progressively converging toward the combined use of radiotherapy and ICIs. A retrospective study by Lehrer et al. [127] compares the safety profiles of concomitant and non-concomitant (separated by 4-week intervals) use of SRS and ICI and portrays manageable risks associated with radiation necrosis (RN). The authors emphasize the importance of limiting the volume of normal brain tissue exposed to radiation doses of 12 Gy or more (V12) to improve radiation dose uniformity and subsequently to reduce the risk of RN. Besides, Rothermundt et al. [128] highlights an illustrative case of a middle-aged woman with multi-organ metastasized clear cell RCC. Transition to pembrolizumab following the induction of RN by whole-brain radiotherapy led to complete disappearing of lung metastases, reducing of BM and stabilization of other areas of metastases.

A pivotal trial (NCT02231749) corroborated the notable efficacy and enduring benefits for patients conferred by the amalgamation of nivolumab and ipilimumab in treating advanced RCC [129]. However, no significant difference was observed in patients experiencing only brain progression between the sunitinib group [130]. The advantages of immunotherapy versus targeted therapy have not been clearly defined.

Despite the auspicious inferences from some prospective studies underscoring the substantial potential of ICIs in RCC BM therapy (as elucidated below), the exigency for more robust evidence from expansive clinical trials remains a persistent narrative [131].

##### BCBM

ICIs still demonstrate limited effectiveness in treating BCBM, with surgery, radiotherapy and molecular subtypes-based targeted therapy and chemotherapy remaining the primary treatment option [14, 132, 133]. Despite the exclusion of BCBM patients in current clinical trials evaluating immune checkpoint in breast cancer, preclinical studies targeting the TME of BCBM provide promising prospects for future ICI strategies.

Findings by Duchnowska et al. [134] showed that PD-L1 and PD-L2 are positively expressed in BCBM at ratios of 53% and 36% respectively, recognizing them as potential targets for ICI. Intriguingly, a positive correlation was found between PD-1 expression in TILs and the presence of CD4 + and CD8 + T cells. This underscores the potential for checkpoint inhibition, even in the face of a perceived negative prognosis commonly associated with such patients who typically demonstrate better control of extracranial diseases and overall systemic conditions.

In a separate study by Griguolo et al. [81] a higher proportion of CD4 + FoxP3 + /CD8 + cells within the HR + /HER2–metastatic tumor stroma was linked with worse outcomes in different breast cancer subtypes. The adverse effect of the PD-1/PD-L1 interaction between immune cells and the tumor stroma on prognosis still suggests potential for ICI despite these findings. Furthermore, Sobottka et al. [135] recognized a relationship between the expression of lymphocyte-activating gene-3 (LAG-3) and PD-1 with an “inflammatory” phenotype commonly found in BCBM, further supporting dual immune checkpoints suppression strategies.

Conventionally, only PD-1/PD-L1 positive BCBM patients were considered for immune checkpoint inhibition. Recent findings from a prospective study (NCT02563925), however, suggest that PD-1/PD-L1 negative patients might also benefit from ICIs [116]. In the same study, although the combined use of tremelimumab (anti-CTLA-4 antibody) and radiotherapy performed modestly in HER2- BM patients, one patient (17%) showed partial response to the combined regimen of tremelimumab, trastuzumab and radiotherapy (HER2 +) for over six months, encouraging further research in this area [136].

In relation to the LAG-3 checkpoint, a clinical trial (NCT00349934) on IMP321, a soluble fusion protein form of LAG-3, exhibited promising results for metastatic breast cancer (including BCBM) [137]. Here, combining paclitaxel with the drug encouraged the proliferation of tumor-killing cells through antigen-presenting cells activation, with a significantly improved objective tumor response rate compared to historical controls (50% vs. 25%). However, the theory adopted in the above study assumes that the ligand of LAG-3 is MHC II, while recent research has promulgated that the main ligand should be fibrinogen-like protein 1, which is upregulated in cancer patients and leads to the unsustainable use of anti-PD-1 therapy [138]. This novel checkpoint pathway reveals great potential for future oncological research, particularly in the direct blocking strategy.

##### Other types of intracranial metastases

Therapeutic advantages have been noted in the combined use of nivolumab and radiotherapy for hepatocellular carcinoma (HCC) BM [139]. The KEYNOTE-177 trial (NCT02563002) attests to the activity of pembrolizumab as the primary treatment for metastatic colorectal cancer [140]. However, specific research aiming at BM from colorectal cancer is in relatively early stages.

Investigations into leptomeningeal metastases (LM), a specific subtype of CNS metastatic tumor, have been ongoing for some time. In a retrospective study on melanoma LM with a historically low overall survival (OS) rate of just 2 months, the use of BRAF/MEK inhibitors or ipilimumab resulted in a significantly higher OS rate than the overall cohort (21.7 weeks vs. 6.9 weeks) [141]. Specifically, the median survival in the group of 10 patients given ipilimumab was 15.8 weeks (range 2–235 weeks, 47 weeks in the RT group and 6 weeks in the non-RT group). This represents an improvement of almost 4 months from the 2.9 weeks OS reported in untreated LM patients. A positive trend is also highlighted in a Phase II clinical trial (NCT03091478) testing pembrolizumab for multiple solid tumors, including NSCLC, head/neck or skin squamous carcinoma, breast cancer and glioma [142]. This trial reported a median intracranial progression-free survival (mPFS) of 2.9 months and a median OS (mOS) of 4.9 months, with intracranial responses seen in 38% of patients treated with ICIs. Of particular note is the achievement of complete intracranial responses in two patients (NSCLC, skin squamous carcinoma). The primary implications of these findings suggest promising avenues for future exploration of ICIs specific to LM from melanoma and other primary tumors demonstrating immunotherapy sensitivity.

#### Combined therapy

Combined therapy presents a compelling approach in the immunotherapy of BM, particularly the administration of ICIs in conjunction with radiotherapy post-surgical resection. A wealth of data and analyses substantiate the safety and efficacy of this integrative approach combining radiation and immunotherapy [143, 144].

Wasilewski et al. [145] investigated on 1690 German NSCLC patients spanning January 2010 to December 2021, of whom 384 were eventually enrolled. The study found that the mOS of patients who received a combination of radiotherapy and ICIs following the surgical resection of intracranial lesions was significantly superior compared to that of individuals treated with postoperative radiotherapy and chemotherapy (23.0 months vs. 11.8 months, *p* < 0.001). In an earlier retrospective analysis (2010–2016) by Chen et al. [146] which surveyed patients with brain metastatic NSCLC, melanoma and HCC, it was observed that patients who were concurrently administered SRS and ICI (within a two-week interval) experienced substantial survival benefits, with no correlation to an increase in the incidence of adverse events (AEs). In another study that specifically investigated the combined treatment of ICIs and SRS for MBM), Lehrer et al. affirmed the safety of this approach, recommending control of V12 (defined in this context to include the therapeutic target area, a differentiable departure from previously employed volume definitions) to below 10 cm^3^.

The efficacy of concurrent therapy, in which radiotherapy and ICIs are administered within a brief interval (specific durations differ across various studies), has been examined. In a study by Qian et al. [147] it was found that among 110 patients with BM from NSCLC or melanoma, concurrent therapy led to a higher response rate (70% vs. 47%, *P* < 0.001) and a lower disease progression rate (5% vs. 26%, *P* < 0.001), compared to nonconcurrent treatment. Furthermore, the meta-analysis findings of Lehrer et al. [148] indicated that when compared to nonconcurrent use of SRS and ICI, the concurrent strategy yielded superior 1-year OS rates (64.6% vs. 51.6%, *P* < 0.001) for patients with BM from Melanoma, NSCLC, or RCC. However, a retrospective study conducted by Helis et al. [149] revealed that the cumulative two-year incidence of adverse radiation effects in patients who received ICIs post-SRS was significantly higher than in patients who didn’t receive ICIs post-SRS (4.5% vs. 2.1%, *P* = 0.004). Herein, intracranial transfer volume and V12 were correlated with risk. Although potential confounding factors such as shorter survival time in the ICI-naive group and misclassification of pseudo-progression into radiation necrosis are present, the study suggests a cautious approach so as to accurately determine the safety of this promising strategy.

Moreover, the sequence of radiotherapy and ICI administration could also be crucial. Pomeranz et al. [150] demonstrated that for patients with MBM who underwent surgical resection, better survival outcomes were observed when RT was administered prior to ICI treatment, as opposed to the reverse sequence. This observation could likely be attributed to the premise that initial RT creates an immune microenvironment conducive for CD8 + T cells.

The efficacy and safety of combining ICIs with platinum-based chemotherapy has gained substantial attention [151–153]. A retrospective analysis by Powell et al. [154] on data from three prospective trials (NCT02039674, NCT02578680 and NCT02775435) focused on stable NSCLC BM patients treated with pembrolizumab plus platinum-based chemotherapy. The study found that the mOS (18.8 months vs. 7.6 months), mPFS (6.9 months vs. 4.1 months), objective response rate (39.0% vs. 19.7%) and median duration of response (11.3 months vs. 6.8 months) were all superior in the ICI plus chemotherapy group compared to the chemotherapy-alone group. Additionally, the incidence of AEs related to the combination therapy was found to be similar to platinum therapy (88.2% vs. 82.8%) for patients with BM [154]. Besides, Japan has initiated a phase II prospective clinical trial (jRCTs071210019) in May 2021, which encompasses the combination of nivolumab + ipilimumab and platinum-based chemotherapy for NSCLC BM, and the outcomes are worthy of close attention in future [155].

The incorporation of LITT or ultrasound therapy also holds promising potential in combination treatment strategies. The therapeutic effect of intracranial ICIs has been associated with the presence of extracranial tumors. For instance, Taggart et al. [156] observed that intracranial anti-PD-1/anti-CTLA-4 activity depends on CD8 + T cells, which are sensitized by ICIs extracranially and enter the brain parenchyma via recruitment. Consequently, devising methods to modify recruitment channels and the microenvironment to accommodate the immune effects of T cells has emerged as a novel concept. For instance, LITT can not only leverage its thermal effects to kill tumors but can also enhance the effects of ICI by temporarily increasing the permeability of the BBB and enhancing local immune function [157]. Similarly, ultrasound therapy is anticipated to remodel the TME by stimulating APC activation, enhancing cytotoxic cell infiltration, guiding macrophage proinflammatory transformation and downregulating immunosuppressive cells. In addition, the temporary permeability of the BBB fosters antitumor cell recruitment and other transcranial delivery processes [158].

#### Prospective clinical trials

Though a substantial amount of retrospective data has indeed demonstrated the formidable antitumor potential of ICIs within the realm of immunotherapies, it’s also essential to acknowledge that more persuasive clinical trial evidence from prospective studies is still critical to achieve comprehensive predictability. Notably, a vast body of literature has reported these meaningful results (Table 1).

**Table 1:** Published prospective clinical trials of ICI therapy involving BM

App	Drug	Team (Ref.)	NCT No.	Phase	Time	Scale*	Add	Tumor Type	Cohorts/Arms (patients)	IR† /ICB‡ (95% CI)	imPFS, months (95% CI)	mOS, months (95% CI)	LOS rates (95% CI)
Single	Pembrolizumab	Goldberg et al. [138]	02085070	II	2008.07–2011.04	36	USA	Mm NSCLC	A: melanoma (13); B: NSCLC (13)	A: 22%† (7–48); B: 33%† (14–59)	–	A: 7.7 (3.5-NR); B: NR	–
	Pembrolizumab	Kluger et al. [139]	02085070	II	2014.03–2015.06	23	USA	Mm	–	26%† (10–48)	2 (2-NR)	17 (10-NR)	48% (31–73) at 2Y
	Pembrolizumab	Goldberg et al. [140]	02085070	II	2014.03–2017.01	42	USA	NSCLC	A: PD-L1 expression ≥ 1% (37); B: PD-L1 expression < 1% (5)	A: 29.7%† (15.9–47.0); B: 0	A: 2.3 (1.9-NR)	A: 9.9 (7.5–29.8)	A: 40% (30–64) at 1Y; 34% (21–54) at 2Y
	Atezolizumab	Gadgeel et al. [142]	02008227	III	2014.03–2018.01	850	Global	NSCLC	Aα: atezolizumab (no BM history, 364); Aβ: atezolizumab (BM history, 61); Bα: Docetaxel (no BM history, 343); Bβ: Docetaxel (BM history, 62)	–	Aα: NR; Aβ: NR; Bα: NR; Bβ: 9.5 (5.8–20.1)	Aα: 13.2 (11.1–15.5); Aβ: 16.0 (10.6–20.1); Bα: 9.3 (8.6–11.1); Bβ: 11.9 (7.0–14.1)	Aα: 31.6% (26.7–36.5) at 2Y; Aβ: 26.6% (15.1–38.1); Bα: 21.4% (16.9–25.9); Bβ: 19.3% (8.2–30.4)
	Ipilimumab	Margolin et al. [141]	00623766	II	2014.03–2018.05	72	USA	Mm	A: asymptoms & no steroid (51); B: symptoms & steroid (21)	A: 18%‡ (8–31); B: 5%‡ (0.1–24)	A: 1.5 (1.2–2.5); B: 1.2 (1.2–1.3)	A: 7.0 (4.1–10.8); B: 3.7 (1.6–7.3)	A: 55% (41–68) at 0.5Y, 31% (18–44) at 1Y, 26% (14–39) at 2Y; B: 38% (17–59) at 0.5Y, 19% (2–36) at 1Y, 10% (0–22) at 2Y
	Pembrolizumab	Naidoo et al. [122]	03091478	II	2016.02–2018.09	13	USA	LM	–	38%†(13.9–68.4)	2.9 (1.3-NR)	4.9 (3.7-NR)	3/13 at cutoff
	Nivolumab	Flippotet al. [149]	03013335	II	2017.02–2019.12	73	FRA	RCC	A: BM untreated (39); B: BM pretreated (34)	A: 11.8%† (3.3–27.5); B: \	A: 2.7 (2.3–4.6); B: 4.8 (3.0–8.0)	/	A: 69.2% (52.2–81.2) at 0.5Y, 66.7% (49.6–79.1) at 1Y; B: 70.6% (52.2–83.0) at 0.5Y, 58.8% (40.6–73.2) at 1Y
Dual	Nivolumab Ipilimumab	Long et al. [143]	02374242	II	2013.01–2018.09	79	AUS	Mm	A: nivolumab + ipilimumab (asymptomatic untreated, 36); B: mono-Nivolumab (asymptomatic untreated, 27); C: mono-Nivolumab (poor prognostic, 16)	A: 46%†(29–63); B: 20%† (7–41); C: 6%† (0–30)	A: NR (2.9–NR); B: 2.5 (1.7–2.8); C: 2.3 (1.4–4.3)	A: NR (8.5–NR); B: 18.5 (6.9–NR); C: 5.1 (1.8–NR)	A: 78% (65–94) at 0.5Y; B: 68% (52–89); C: 44% (25–76)
	Nivolumab Ipilimumab	Tawbi et al. [144]	02320058	II	2014.11–2017.08	94	USA	Mm	/	57%‡ (47–68)	NR	NR	92.3% (84.5–96.3) at 0.5Y; 82.8% (73.1–89.3) at 0.75Y; 81.5% (71.5–88.2) at 1Y
	Nivolumab Ipilimumab	Tawbi et al. [145]	02320058	II	2015.02–2017.11	119	USA	Mm	A: asymptoms (101); B: symptoms and/or steroid (18)	A: 58.4%‡ (48.2–68.1); B: 22.2%‡ (6.4–47.6)	A: NR (6.5-NR); B: 1.2 (0.7–1.3)	A: NR; B: 8.7(8.8-NR)	A: 82.4% (73.2–88.7) at 1Y, 75.2% (64.9–82.8) at 1.5Y; B: 65.8% (39.1–83.0) at 0.5Y
	Nivolumab Ipilimumab	Tawbi et al. [146]	02320058	II	2015.02–2018.05	119	USA	Mm	A: asymptoms (101); B: symptoms and/or steroid (18)	A: 57.4%‡ (47.2–67.2); B: 16.7%‡ (3.6–41.4)	A: 39.3 (7.5–45.8); B: 1.2 (0.7–NR)	A: NR; B: 8.7 (8.9-NR)	A: 71.9% (61.8–79.8) at 3Y; B: 36.6% (14.0–59.8) at 3Y
	Nivolumab Ipilimumab	Giacomo et al. [147]	02460068	III	2015.02–2020.12	80	ITA	Mm	A: mono-fotemustine (27); B: ipilimumab + fotemustine (26); C: ipilimumab + nivolumab (27)	A: 0; B: 19.2%†(4.1–34.4); C: 44.4%† (25.7–63.2)	A: 3.0 (2.3–3.6); B: 3.3 (1.2–5.4); C: 8.7 (0.0–19.9)	A: 8.5 (4.8–12.2); B: 8.2 (2.2–14.3); C: 29.2 (0–65.1)	A: 34.8% (15.4–54.2) at 1Y, 21.7% (4.9–38.5) at 2Y, 16.3% (0.6–32.0) at 3Y, 10.9% (0–24.4) at 4Y; B: 38.5% (19.9–57.1) at 1Y, 19.2% (4.1–34.3) at 2Y, 15.4% (1.5–29.3) at 3Y, 10.3% (0–22.6) at 4Y; C: 66.7% (48.9–84.5) at 1Y, 51.9% (33.1–70.7) at 2Y, 47.9% (28.9–66.9) at 3Y, 41.0% (20.6–61.4) at 4Y
	Nivolumab Ipilimumab	Emamekhoo et al. [150]	02982954	IIIb/IV	2017.01–2021.10	28	USA	RCC	–	32%†(14.9–53.5)	9.0 (2.9–12.0)	NR (14.1-NE)	85.6% (66.0–94.3) at 1Y; 67.0% (46.1–81.3) at 1.5Y; 63.2% (42.4–78.3) at 2Y
	Nivolumab Ipilimumab	Reck et al. [151]	02477826	III	2015.08–2022.02	1739	Global	NSCLC	Aα: dual ICI (with baseline BM, 68); Aβ: ChT (with baseline BM, 66); Bα: dual ICI (without baseline BM, 515); Bβ: ChT (without baseline BM, 517)	–	Aα: 8.6 (5.7–19.5); Aβ: 8.7 (6.6–11.5) [5-years rates:Aα–16%Aβ–6%]	Aα: 17.4(9.2–29.4); Aβ: 13.7(10.5–16.2); Bα: 17.2(15.3–20.0); Bβ: 13.9(11.8–15.3)	Aα: 20% (12–31) at 5Y; Aβ: 6% (2–14); Bα: 23% (19–26); Bβ: 13% (10–16)
co-TT	Tremelimumab -/ + Trastuzumab	Page et al. [116]	02563925	NA	2010.06–2014.07	26	USA	BC	A: HER2- (20); B: HER2 + (6)	A: 15%†; B: 33%†	A: 3.0[range:1.1–6.2]; B: 3.1[range:1.3–8.7]	A: 4.9[range: 1.1–22.8 +]; B: 8.0[range: 1.3–15.1]	–
co-RT	Ipilimumab	Williams et al. [152]	01703507	I	2012.10–2014.08	16	USA	Mm	A: WBRT (5); B: SRS (11)	–	A: 2.5; B: 2.1	A: 8; B: NR	–
co-ChT	Pembrolizumab + Platinum-based ChT	Powell et al. [134]	02039674 02578680 02775435	II/III	Cutoff dates: 2017/2018	1298	Global	NSCLC	Aα: ICI + ChT(with BM, 105); Aβ: ChT (with BM, 66); Bα: ICI + ChT (without BM, 643); Bβ: ChT (without BM, 484)	–	–	Aα: 18.8 (13.8–25.9); Aβ: 7.6 (5.4–10.9); Bα: 22.5 (19.8–25.2); Bβ: 13.5 (11.3–15.8)	Aα: 62.9% at 1Y; Aβ: 34.9%; Bα: 70.2%; Bβ: 53.6
	Ipilimumab + Fotemustine	Giacomo et al. [148]	01654692	II	2015.09–2021.07	86	ITA	Mm	α: the whole (86); β: baseline BM (20)	–	α: 8.3 (4.7–11.8); β: 3.0 (2.9–3.1)	α: 12.9 (7.1–18.7); β: 12.7 (2.7–22.7)	α: 33.4% at 2Y, 28.5% at 3Y; β: 38.9% at 2Y, 27.8% at 3Y

co-TT, combined with targeted therapy; co-RT, combined with radiotherapy; co-ChT, combined with chemotherapy; CI, confidence interval; imPFS, intracranial median progression-free survival; mOS, median overall survival; OS, overall survival; LOS, Long-term overall survival; Y, year. AEs, adverse events; NR, not reached; NA, not applicable; NE, not estimated; Mm, melanoma; NSCLC, non-small cell lung cancer; RCC, renal cell carcinoma; BC, breast cancer; BM, brain metastases; LM, leptomeningeal metastases

*Patients enrolled into cohorts and finally analyzed

†IR, intracranial responses (complete response + partial response)

‡ICB, intracranial clinical benefit [complete response + partial response + stable disease ≥ 12 weeks (just NCT00623766) /6 months]

A portion of these trials aim to corroborate the efficacy of monotherapies using ICIs or combination therapies involving multiple ICIs, which is beneficial for ICIs to qualify as a first-line treatment. For instance, a phase II trial (NCT02085070) that administered pembrolizumab to a selection of 52 specific BM patients between 2014 and 2015 demonstrated response rates of 22% for MBM and 33% for NSCLC BM, with both groups reported to have shown durable responses [159]. Consequently, this trial showcased the efficacy of pembrolizumab in treating untreated or progressive BM via systemic application. It’s worth highlighting that the efficacy advantage of first-line NSCLC chemotherapy combined with pembrolizumab, as observed in the KEYNOTE-189 study (NCT02578680), was equivalently significant in the BM subgroup. The BM subgroup reported mPFS of 6.9 months and mOS of 19.2 months [152]. Unquestionably, these encouraging outcomes support the application of pembrolizumab for treating BM.

Even more importantly, the long-term follow-up results based on the aforementioned clinical study (NCT02085070) further confirmed the potential of pembrolizumab: (i) For MBM patients, the mPFS was 2 months, the mOS was 17 months, and the 2-year survival rate stood at 48%. Most neurotoxicity was classified as grade 1/2, indicating acceptable safety [160]. (ii) For the cohort of NSCLC BM patients with PD-L1 expression ≥ 1%, the response rate and 2-year survival rate were 29.7% and 34% respectively. The incidence of serious treatment-related AEs was 14% (6/42), and there were no treatment-related deaths [161]. Additionally, a phase III clinical trial (NCT00623766) showcased the disease control activity of ipilimumab in stable, asymptomatic MBM patients without steroid therapy [162]. Another phase III clinical trial (NCT02008227) demonstrated a trend of OS benefit in asymptomatic NSCLC BM patients treated with atezolizumab compared to docetaxel [mOS: 16.0 vs. 11.9 months; Hazard Ratio (HR): 0.74; 95% Confidence Interval (CI) 0.49–1.13] [163].

The exploration of combination therapy has likely gained more popularity in clinical practice than single ICI application. For instance, a seminal multi-center phase II clinical trial (NCT02374242) conducted from 2014 to 2017 demonstrated the potential of nivolumab combined with ipilimumab for first-line treatment of asymptomatic and non-responsive MBM without local radiation therapy (with an intracranial complete response rate of 17% in the combination cohort, surpassing other monotherapy cohorts) [164]. Similarly, another phase II study (NCT02320058) exhibited comparable clinical benefits and objective response rates for the combination therapy of nivolumab and ipilimumab in treating asymptomatic and untreated MBM (57% vs. 56%; 55% vs. 50%), thereby validating the clinical significance of dual ICI therapy for stable MBM [165].

The CheckMate 204 trial (NCT02320058) went a step further and reported that the median duration of response, PFS and OS for dual ICIs therapy for asymptomatic MBM had exceeded the follow-up time (20.6 months). It was consequently recommended that the immunotherapy standard for asymptomatic MBM patients should be established as 1 mg/kg nivolumab + 3 mg/kg ipilimumab [166]. The final 3-year follow-up subsequently confirmed the significance of combined therapy for asymptomatic MBM. It prompted the development of a strategy to discontinue cortisol prior to the initiation of immunotherapy and explore the combination with novel ICIs or adjunctive strategies such as surgery and SRS [167]. Further, a more comprehensive 4-year phase III trial (NCT02460068) substantiated that nivolumab in conjunction with ipilimumab brought about more considerable survival benefits for asymptomatic MBM patients compared to fotemustine [168]. Contrastingly, the NIBIT-M1 trial (NCT01654692) brought to light that formustine does not seem to interfere with the immune activity of ipilimumab, implying that the combination of ipilimumab and fotemustine could potentially represent an effective treatment strategy for MBM patients [169].

In addition to MBM, existing prospective clinical trials (NCT03013335, NCT02982954) have also demonstrated that strategies involving nivolumab or a combination of nivolumab with ipilimumab can also benefit RCC patients with BM [170, 171]. Moreover, a review of the follow-up outcomes from the CheckMate 227 trial (NCT02477826) provides further evidence for the combination of nivolumab and ipilimumab in the first-line therapy of NSCLC patients with baseline BM, noting longer OS (HR: 0.63; 95% CI 0.43–0.92) and higher 5-year intracranial PFS rates (16% vs. 6%) when compared to chemotherapy [172].

Other trials have explored the combination of ICIs and standard treatments such as surgery and radiotherapy, further promoting the clinical application of immunotherapy. For instance, a phase I trial (NCT01703507) demonstrated the safety of using 3 mg/kg or 10 mg/kg ipilimumab in combination with SRS in MBM patients [173]. Another trial (NCT02563925) underscored the clinical significance of tremelimumab for HER2 + breast cancer patients with BM who received combined radiotherapy and trastuzumab [136].

Besides, the importance of imaging evidence to evaluate the therapeutic effect and safety of BM, including amino acid PET, FDG PET, PWI, DWI and MRI, has been highly valued [174]. For example, in a study (NCT03520634) of nivolumab plus ipilimumab in MBM patients, Nienhuis et al. [175] demonstrated that PET imaging using 18F-BMS986192 as a tracer could effectively predict tumor shrinkage caused by ICIs, correlate changes in lesion size during follow-up and detect treatment-related toxicity.

### CAR-T Cells

#### Mechanism

The study of CAR-T cells, particularly in the field of tumor therapy, has witnessed significant advancements since 1989, when Eshhar et al. [176] successfully generated antibody-like specific T cells with chimeric receptors through the transfection of engineered genes into a cytotoxic T cell hybridoma. Activation of natural T cells requires the TCR binding to peptide-MHC complex and antigen as the primary signal, in addition to the costimulatory signal involving the molecule CD28 binding to B7 [177].

**Fig. 3:**
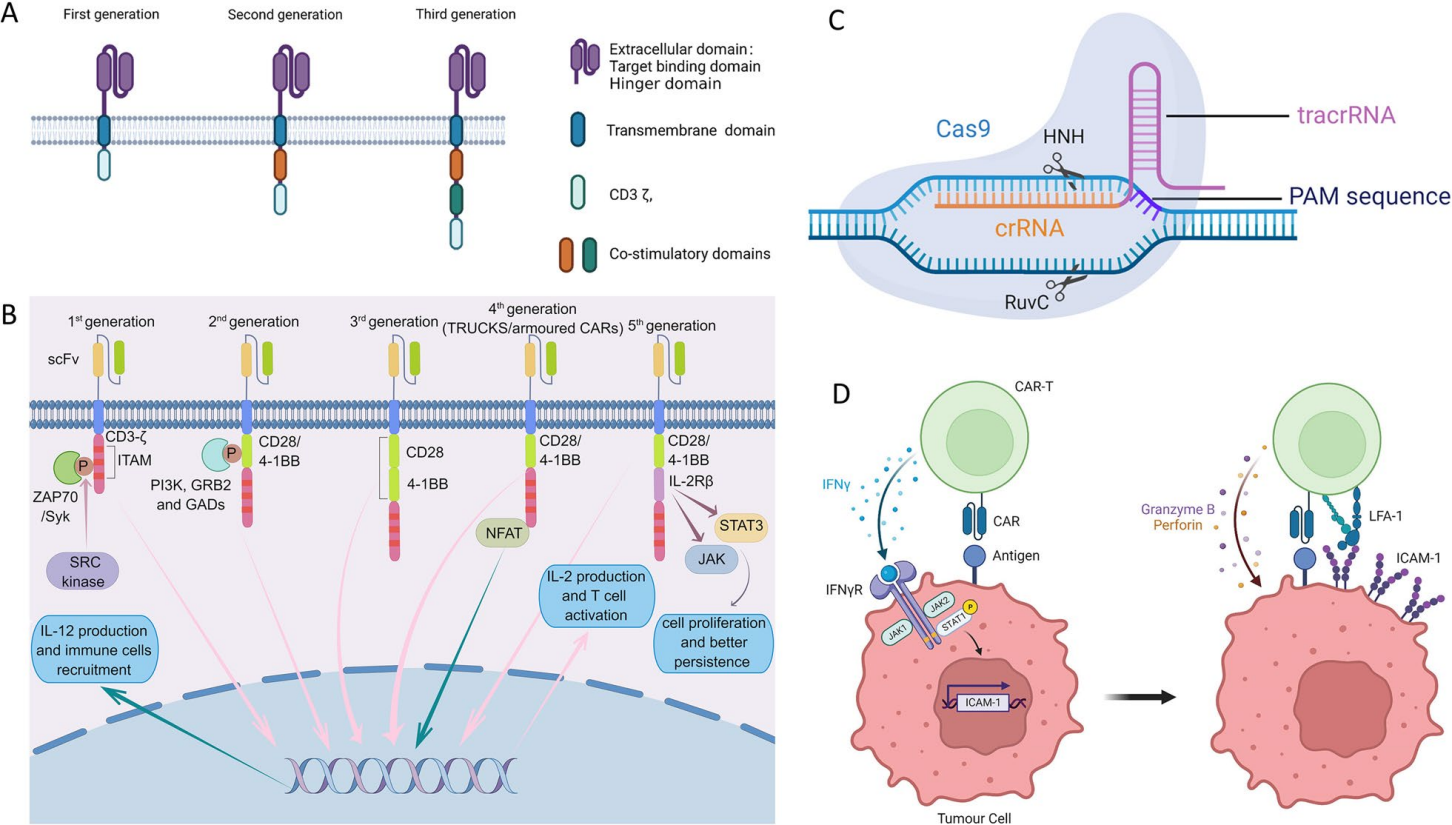
CAR structure and stimulation mechanism. **A** The basic structure of CARs mainly consists of four sections: (i) the target binding domain, linked to the intracellular section by the hinger domain, is located extracellularly to recognize the specific antigen on the tumor surface; (ii) the transmembrane domain; (iii) the CD3ζ signaling domain; and (iv) the costimulatory domain, mainly the molecule 4-1BB or CD28, which encourages intracellular transduction of stimulatory signals. Reproduced under the terms Creative Commons Attribution 4.0 International License (CC BY 4.0) (https://creativecommons.org/licenses/by/4.0/)[178]. Copyright 2022, The Authors, published by BioMed Central. **B** CAR activates the second messenger molecules in the tyrosine kinase signaling pathway by cascading phosphorylation after identifying TAAs on the surface of tumors, thus stimulating intranuclear genes to express the inflammatory factor IL-2 and promote T cell activation. Furthermore, the fourth generation utilizes NFAT to stimulate inflammatory factor IL-12 production and recruit more immune cells, and the fifth generation utilizes the JAK-STAT pathway to prove T cell proliferation and achieve better persistence. Reproduced under the terms CC BY 4.0 (https://creativecommons.org/licenses/by/4.0/) [179]. Copyright 2022, The Authors, published by BioMed Central. **C** CRISPR/Cas9 is a popular tool for editing CAR-T cell genes: crRNA supports target sequence identification; tracrRNA supports structural stability of the complex; protospacer adjacent motif (PAM) sequence assists in locating the target sequence; HNH and RuvC nuclease domains cut target and nontarget DNA strands, respectively. Reproduced under the terms CC BY 4.0 (https://creativecommons.org/licenses/by/4.0/) [181]. Copyright 2022, The Authors, published by BioMed Centra.** D** CAR-T cells release the inflammatory factor IFNγ, stimulating the tumor cell IFNγR signaling pathway, which mediates intercellular adhesion, is critical in the interaction between CAR-T cells and solid tumors and ultimately ensures the cytotoxicity of granzyme B and perforin. Reproduced under the terms CC BY 4.0 (https://creativecommons.org/licenses/by/4.0/) [210]. Copyright 2022, The Authors, published by Frontiers

In addition to the two key factors, CARs have enabled the artificial modification of T cells, enhancing their ability for targeted activation. The extracellular antigen portion of CAR serves as the binding domain that recognizes specific tumor-associated antigens (TAAs). It is structurally composed of a variable heavy chain (VH) and variable light chain (VL) and connected to the transmembrane domain by spacers (or hinge region) derived from CD28, CD8 or IgG to ensure its flexibility (Fig. 3A) [178, 179].

The intracellular signaling domain is crucial for determining whether the stimulus signal is adequate to induce the desired cellular response. As a result, first-generation CARs only feature a single CD3ζ signaling domain, but their limited signal persistence has yielded unsatisfactory clinical outcomes (Fig. 3B) [179, 180]. Building upon the first generation, the second and third generations have incorporated single or double costimulatory domains, respectively. These advancements primarily involve the integration of the CD3ζ signal domain, along with costimulatory domains consisting mainly of 4-1BB or CD28, into the current CAR design (Fig. 3A) [178, 180, 181].

Lu and Jiang have summarized the progress made in the design of the two newer generations of CARs [179]. The aim of these designs is to enhance T cell recruitment or sustained activation through intracellular signal transduction. In the second generation, T cells are engineered to express and secrete cytokines, promoting T cell activation. Furthermore, the fourth generation (known as TRUCKs or armored CARs) induces IL-12 production by activated nuclear factor of activated T cells (NFAT), while the fifth generation enhances cell proliferation and prolongs persistence by driving the JAK-STAT pathway with IL-2 receptor β [182, 183].

#### Development: from Leukemia to BM

Since August and October 2017, when the US Food and Drug Administration (FDA) approved the pioneering drugs Tisagenlecleucel and Axicabtagene Ciloleucel [184, 185], CAR-T cells have established themselves as a substantial therapeutic strategy in the fight against cancer [179, 186]. As of March 2022, there are eight approved drugs worldwide; however, their indications are primarily confined to hematological malignancies, including B cell acute lymphoblastic leukemia, large B cell lymphoma, follicular lymphoma, mantle cell lymphoma and multiple myeloma. In 2013, the first registered clinical trial (NCT01735604) for CAR-T therapy in China was initiated by the PLA General Hospital team [187]. The trial aimed to target CD20 in combatting against diffuse large B cell lymphoma [188]. Presently, China has registered over 700 clinical trials, reflecting alignment with international development trends [187].

Research on solid tumors, mainly hepatic carcinoma, pancreatic cancer and glioma, is increasing year by year [187]. Nevertheless, the challenge of deploying CAR-T cells in solid tumors remains due to three primary reasons [178, 189–191]:

(I) Antigen targeting problem: There is a need for more specific antigens as targets for engineering T cells. Normal cell damage caused by off-target toxicity cannot be quickly compensated, as seen with blood cells.
(II) Maintenance of T cell infiltration and cytotoxicity: Extralymphatic delivery of T cells is a challenge, particularly for neurological tumors, which necessitate overcoming obstacles such as the BBB. Experiments on solid tumors indicate that the persistence of T cells is limited, complicating the achievement of sufficient antitumor effects.
(III) Impact of the TME: The heterogeneity of the TME necessitates more intricate design consideration for CARs, while the complex immune microenvironment may potentially inhibit the immunological impacts of CAR-T cells.

In solid tumors, the complex TME exhibits profound implications on CAR-T cell therapy at physical, metabolic and immune levels. Within the tumor, elevated solid stress, increased interstitial fluid pressure, enhanced stiffness and abnormal matrix microarchitecture elevate the difficulty of CAR-T cells physically infiltrating the TME. The compaction and remodeling of the extracellular matrix (ECM) microarchitecture in particular heightens the tumor’s intrinsic fluid pressure (IFP) and macroscopic stiffness, thereby impeding the interaction between CAR-T cells and tumor cells [192]. Cao et al. [193] demonstrated that microwave ablation (MWA) could alleviate high levels of hyaluronic acid present in the tumor ECM, and by provoking stronger blood flow, reducing IFP and elevating oxygen content, they were able to enhance tumor permeability. This, in turn, promoted the infiltration and accumulation of targeted AXL CAR-T cells in AXL-positive NSCLC models, thus producing exceptional antitumor activity. Furthermore, FUS can also remodel the ECM via its thermal and biomechanical effects, assisting in the infiltration of engineered T cells and circumventing immune suppression interference [194]. The combination of a nanomaterial delivery system and CAR-T therapy also provides a novel approach to overcoming the physical barrier of the TME [190].

The uncontrolled proliferation of tumor cells and the inefficient energy transformation induced by the Warburg effect result in a hypoxic and malnourished TME [195] This poses a unique metabolic barrier for CAR-T therapy. Strategies proposed by Gao et al. [196] to overcome these challenges include combining antiangiogenic therapy, in vitro hypoxic environment pretreatment and bioengineering to stimulate the overexpression of key enzymes, thereby enhancing T cell glycolytic activity.

The TME is heavily reliant on immunosuppressive cells, such as myeloid-derived suppressor cells (MDSCs), tumor-associated macrophages (TAMs), regulatory T cells (Tregs) and regulatory B cells (Bregs). Immune inhibitory factors, including proinflammatory cytokines, immune checkpoint molecules, C-X-C subfamily chemokines and their receptors, C–C subfamily chemokines, growth factors and reactive oxygen species, together form a complex tumor immune microenvironment. This, in turn, interferes with the intratumoral invasion, accumulation and immune effect of CAR-T cells [197].

To tackle these obstacles, Ma et al. [198] designed a chimeric antigen vaccine with albumin affinity that can be delivered to lymph nodes and then modify the surface of antigen-presenting cells, thus altering the simulated location of CAR-T cells to the lymphatic microenvironment for proliferative expansion, ultimately enhancing the inhibitory effect on mouse solid tumor models. Additionally, Wang et al. [199] used oncolytic adenoviruses (oAds) that express the chemokine CXCL11 to upregulate tumor suppressive cells in the TME, downregulate immunosuppressive cells like Tregs and MDSCs and promote the transition of macrophages from anti-inflammatory M1 polarization to proinflammatory M2 polarization. This effectively remodeled the TME, thus allowing B7H3. CAR-T cells loaded with CXCL11-armed oAds to achieve sustained inhibition of glioblastoma (GBM) growth when combined with intratumoral administration.

In fact, GBM can be seen as the forefront of CAR-T cell application research in solid tumors. It provides invaluable models and directions for the study of BM, which are also CNS tumors.

#### Advances for BMs

CAR-T (Chimeric Antigen Receptor T cell) therapy is rapidly expanding its scope of application from hematological malignancies to BM, notably specific areas of solid tumors in the central nervous system, which demonstrates its tremendous potential. This cutting-edge immunotherapy, including CAR-T cells, has shed new light upon the future of refractory tumors. Notably, it provides a potential solution for the persistent uncertainties surrounding the positive margins of surgical resection and radiotherapy for intracranial tumors [200]. Currently, a phase 1 clinical trial (NCT03696030) of HER2-targeted CAR-T cells in the treatment of HER-2-positive breast cancer patients with recurrent brain or leptomeningeal metastases is in progress, and other basic frontier experiments regarding BM have also been carried out (Table 2).

**Table 2:** Laboratory advances in the CAR-T brain metastases therapy

Team	Year	Region	Tumor Type	Cell Line	Tumor Model	CAR	Contrast	Application	Results	Ref.
Priceman et al.	2018	USA	Breast	Human breast cancer	Xenotransplant models in mice	HER2-BBζ	HER2-28ζ Mock	In vitro i.c i.c.v	HER2-BBζ: cytokine production** ↓**, T cell exhaustion phenotype **↓**, proliferative capacity **↑**; i.c.: significant antitumor responses, extend survival; i.c.v.: treating multifocal BM evidence	[180]
Li et al	2022	CHN & USA	Lung	Human NSCLC	Xenotransplant models in mice	B7-H3 co-expressed CCR2b	B7-H3 without co-expression	Intravenous	significant antitumor activity; more tumor-specific enhanced infiltration	[170]
Subham et al	2022	USA	Breast	Human TNBC	Xenotransplant models in mice	EGFR806	Mock	In vitro i.c	effectively kill tumor in vitro; i.c.: effective tumor regression, statistically significant survival	[181]
Xu et al	2023	DEU	Lung	Murine Lewis Lung carcinoma	Intraparenchymal models in mice	Anti-EpCAM	–	Local vs i.v	local: significant decreased brain tumor growth; i.v.: no anti-tumorous evidence;	[182]

NSCLC, non-small cell lung cancer; TNBC, triple-negative breast cancer; i.c., intratumoral/intracranial; i.c.v., intraventricular/intracerebroventricular; i.v., intravenous

Li et al. [191] utilized bioengineered B7-H3 CAR-T cells co-expressing the chemokine receptor CCR2b, targeting the high expression of the chemokine CCL2 in both primary NSCLC and NSCLC BM. Their findings demonstrated a higher level of tumor-specific infiltration in comparison to the non-coexpressing group. Therefore, they succeeded in leveraging the specificity of the TME to significantly improve the antitumor capability of engineered T cells.

The study by Priceman et al. [201] underscored the importance of CAR design in terms of tumor target specificity. They demonstrated that: (i) HER2-targeted CAR-T cells with 4-1BB costimulatory signaling domain exhibited fewer T cell failure phenotypes and stronger proliferative ability compared to cells containing CD28 costimulatory domain; (ii) in terms of application methods, controlled trials have confirmed that local intratumoral application achieves more effective antitumor performance compared to intravenous application; and (iii) moreover, the team’s research also provided laboratory evidence substantiating the efficacy of CAR-T in treating multifocal BM and leptomeningeal disease via intraventricular injection.

Subham et al. [202] are currently conducting a phase 1 clinical trial (NCT03638167) on the intracavitary or intraventricular delivery of CAR-T cells for pediatric primary central nervous system tumors which target EGFR806, illustrating its potential applicability to BM stemming from triple-negative breast cancer.

Xu et al. [203] explored the effect of application methods on the performance of CAR-T cells in treating lung cancer BM and concluded that, compared to intravenous injection, local application of EpCAM-CAR-T cells achieved a more significant accumulation in the target tumor and significantly stimulated antitumor effects without causing central nervous system or systemic off-target toxicity. Nevertheless, the researchers found that CAR-T cells lacked sufficient persistence in tumor aggregation and failed to consistently exert tumor cell killing effects.

Overall, the existing literature suggests that the application of CAR-T therapy in the field of BM necessitates careful attention to numerous factors including tumor target selection, CAR costimulatory domain design, co-expression of chemokine receptors and adjunctive, intracavitary or intraventricular delivery applications.

In addition to the specialized experiments mentioned above, more achievements in the antitumor application of CAR-T cells can also inspire further exploration into BM therapy.

The interaction between PD-1, expressed by CAR-T cells, and tumor cell surface ligands often leads to T cell exhaustion. Several strategies have been suggested for addressing this issue: combining ICIs, designing CAR-T cells to produce blocking antibodies against PD1/PD-L1, or utilizing CRISPR-Cas9 to knock out the gene expressing PD-1 in the CAR-T cell nucleus [179, 200]. Simultaneously, genetic engineering technology based on CRISPR‒Cas9 has emerged as a critical tool for the evolution of the CAR-T medical industry. It assists in achieving universality, overcoming off-target toxicity and boosting T cell persistence (Fig. 3C) [181].

Nanomaterial technology has introduced an innovative approach for the application of CAR-T cells in solid tumors, mainly enhancing therapy efficacy in three distinct aspects: T cell tumor targeting and accumulation, survival, proliferation in the TME and delivery systems. For instance, magnetic nanoclusters can combine with CAR-T cells to guide cell targeting through an external magnetic field. Nanoparticle-conjugated CAR-T cells have the capacity to remodel the microenvironment via photothermal or nanoenzyme catalysis, thereby amplifying T cell tumor accumulation and promoting cell survival and proliferation. Nanomaterials can also synergistically deliver antagonists of T cell growth inhibitory factors or immune checkpoint inhibitors, and nanofusion vaccines can enhance antigen presentation and CAR-T cell proliferation. Alternatively, nanomaterials can be directly used to conceive micro scaffolds with T cell growth promoting factors, hence establishing a microenvironment for in vivo amplification of CAR-T cells. Furthermore, nanogel or mRNA nanocarrier delivery systems will also provide a novel research mentality [114, 189, 190, 204].

It’s noteworthy that the era of nanomaterial-assisted CAR-T therapy has commenced within the field of glioblastoma, a central nervous system tumor alike to BM. Wu et al. successfully utilized iron oxide nanoparticles, which were used as MRI contrast agents in another phase II clinical trial (NCT03407495), for imaging tracking of CAR-T cells in a glioblastoma animal model. This could potentially provide key insights for constructing an evaluation system for CAR-T therapy of BM [205, 206]. Furthermore, Ogunanaike et al. [207] delivered B7-H3-targeted CAR-T cells to the surgical margin of glioblastoma using a nanometer hydrogel package, which enhanced cell retention and gradual release, leading to exceptional antitumor effects. This delivery system might act as a valuable reference in future research on nanomaterial-modified CAR-T therapy for BM.

Meng et al. [194] deployed a heat shock protein promoter-associated acoustic-thermal effect-dependent Cre gene switch to manage spatiotemporally restrictive activation of engineered T cells by FUS. This approach enhanced the targeting and controllability of CAR-T cells, effectively inhibiting the growth of subcutaneous tumor models and reducing off-target toxicity. Given the advancements of FUS in effectively penetrating the skull and treating brain tumors [208], acoustic-controlled CAR-T cells with a FUS delivery system could also catalyze the innovation of brain metastases therapy [209].

In existing research, scholars have found that CAR-T cells secrete IFNγ, which stimulates the IFNγR-JAK-STAT pathway in solid tumor cells and thereby elevates the expression of the adhesion molecule ICAM-1. This enhances the adhesion between CAR-T cells and tumor cells, mediating the tumor-killing effect (Fig. 3D) [210]. Moreover, Larson et al. [211] also discovered that IFNγR signaling plays a critical role in the tumor affinity of CAR-T cells in their experiments on glioblastoma. Further research is needed to determine whether this adhesive property also affects metastatic solid tumors of the central nervous system.

Moreover, managing, monitoring and treating the toxicity of immune therapy, such as cytokine release syndrome, immune effector cell-associated neurotoxicity syndrome (ICANS), cytopenia and other immunotherapeutic toxicities without affecting the therapeutic efficacy of CAR-T cells will also be a focus area of exploration. In particular, distinguishing between ICANS and the neural damage caused by brain metastases themselves is incredibly important [212–214].

In summary, the innovative trends for next-generation CAR-T treatments for BM might originate from research combining ICIs, nanomaterial delivery systems, focused ultrasound regulation, or exploring prevention and control of neurotoxic side effects.

### Glial cell targeting strategy

#### Astrocyte

Astrocytes, which are crucial glial cells that mediate the immunosuppressive TME, have become a focal point in immunotherapy due to their role in promoting metastases (Table 3). The STAT3 signal is widely recognized as evidence of the specialized anti-inflammatory phenotype of astrocytes that assist BM malignant cells in colonizing intracranially [26, 96]. STAT3 + astrocytes resist infiltration of CD8 + T cells and upregulate the level of metastases supporting CD74 + Iba1 + microglia/macrophages. The interaction between CD74 and macrophage migration inhibitory factor (MIF), which is secreted by STAT3 + astrocytes, increases the expression of the growth factor midkine. Midkine is a transcription factor that promotes the growth of BM (Fig. 2) [26]. Therefore, astrocyte targeting strategies based on the inhibition of STAT3 are gradually gaining traction.

**Table 3:** Summary of selected BM therapy strategy targeting glial cells

Targeting	Application	Tumor type	Objects	Status	Ref.
Astrocytes					
STAT3	Silibinin	Human and mouse BM cell lines, lung cancer	Mice model, Human Patients	Published in 2018	[81]
	Silibinin	NSCLC, BC	Clinical trial	Recruiting	NCT05689619
	WBRT + silibinin	BM, LC	Clinical trial	Recruiting	NCT05793489
Gap junction	carboplatin + meclofenamate/tonabersat	TNBC, NSCLC	Mice model	Published in 2016	[84]
	T-M/siRNA	BC	Mice model	Published in 2022	[201]
	Meclofenamate	Solid tumor	Clinical trial	Active, non-recruiting	NCT02429570
Microglia/Macrophages					
MRC1 + cells	Mannosylated clodronate liposomes	BC	Mice model	Published in 2017	[28]
PI3K	BKM120	BC	Mice model	Published in 2018	[94]
TLR9	CpG-C*	Lung cancer, melanoma	Mice model	Published in 2019	[205]
CSF1R	PLX3397	BC	Mice model	Published in 2021	[202]
	BLZ945 + AC4-130 (CSF2Rβ-STAT5 inhibitor)	BC	Mice model	Published in 2021	[203]
Estrogen-STAT3	Tamoxifen	BC	Mice model	Published in 2021	[204]
IL6-JAK2-STAT3	Fedratinib (JAK2 inhibitor), tocilizumab (anti-IL6R)	NSCLC	Mice model	Published in 2022	[89]

BM, brain metastases; NSCLC, non-small cell lung cancer; BC, breast cancer; TNBC, triple-negative breast cancer; LC, leptomeningeal carcinomatosis; STAT, signal transducer and activator of transcription; MRC1, mannose receptor 1; PI3K, phosphoinositide 3-kinase; TLR9, Toll-like receptor 9; CSF1R, colony-stimulating factor 1 receptor; CSF2Rβ, colony-stimulating factor 1 receptor beta; IL6, interleukin 6; JAK2, Janus kinase 2

*The agonist, the others are inhibitors

**Fig. 4:**
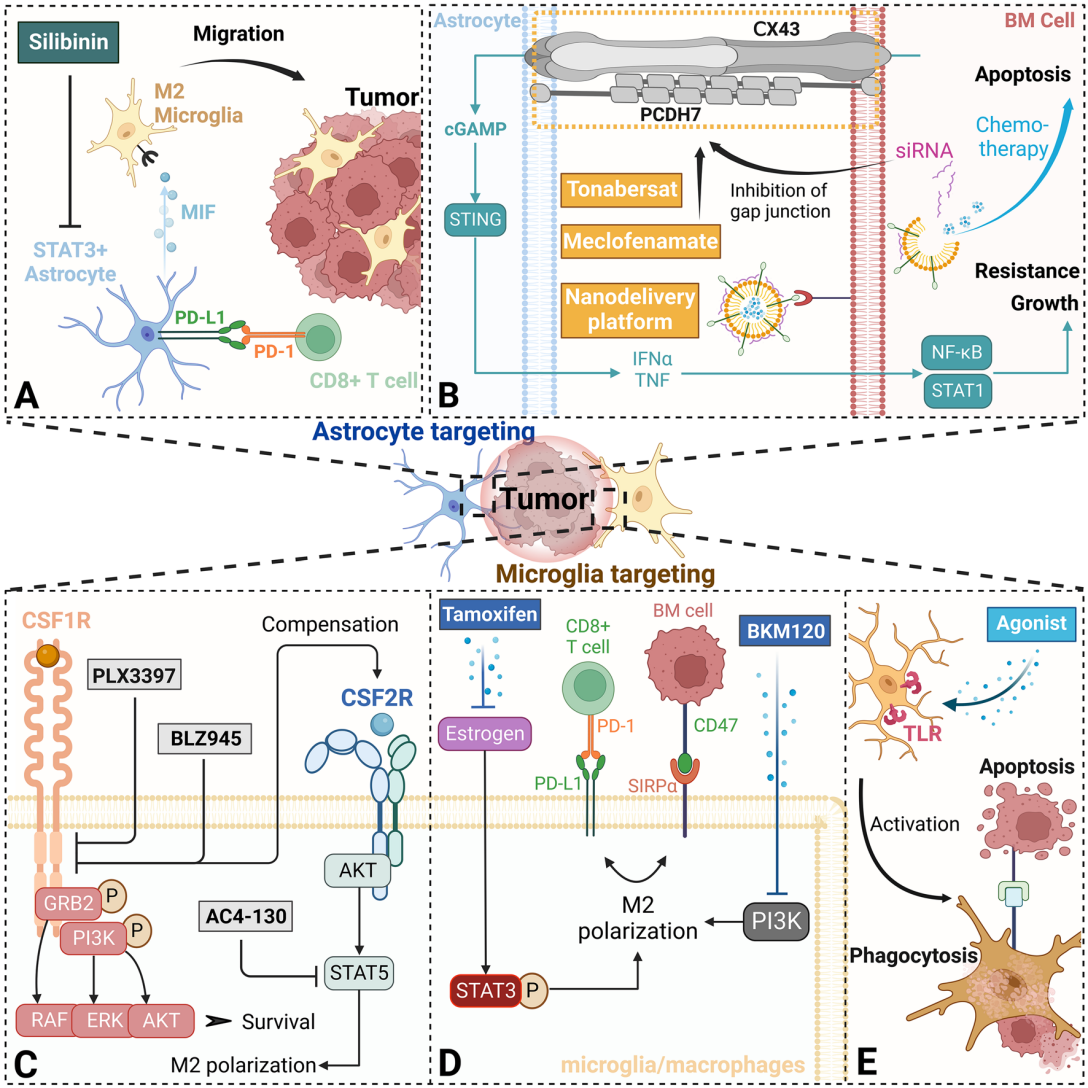
Schematic illustration of BM therapy medicines in glial cell targeting strategies. **A** Silibinin downregulates the STAT3 level in astrocytes, thus suppressing the immune effectiveness of metastases support. **B** The inhibition of the gap junction, which is critical for the metastases-astrocyte crosstalk, although meclofenamate, tonabersat or siRNA is effective in controlling BM. In addition, the construction of nanodelivery platforms such as tailored micelles (T-M/siRNA) have potential for BM therapy. **C** The mechanism of BM-promoting CSF1R signaling with the CSF2R-STAT5 assistance pathway and the action site of the inhibitors, including PLX3397, BLZ945 and AC4-13. **D** Tamoxifen and BKM120, which are traditional targeted drugs, exert immunotherapeutic effects by inhibiting the estrogen-STAT3 pathway and PI3K signaling of protumor microglia, respectively. **E** Diagram of the TLR9 agonist (CpG-C) promoting microglia to clear tumor cells. Created with BioRender.com

Silibinin is a natural compound extracted from the seeds of the milk thistle herb that acts as a downregulator of STAT3 [215]. This substance interferes with the phosphorylation of STAT3 by Janus family kinases (JAKs), SRC tyrosine kinases and ABL tyrosine kinases in tumor cells, inhibiting the formation of STAT3 dimer nuclear signals in tumor cells and thus suppressing the tumor itself [216]. Notably, silibinin also remodeled the STAT3 + astrocyte-dependent metastases, promoting BME (Fig. 4A). Two ongoing clinical trials (NCT05689619 and NCT05793489) are currently using silibinin, either as monotherapy or in combination with WBRT, for the treatment of BM.

In addition to silibinin, WP1066 is another inhibitor of STAT3. Kong et al. [217] used WP1066 to inhibit the JAK2-STAT3 signaling pathway and suppress immunosuppressive FoxP3 + regulatory T cells, achieving therapeutic effects in a melanoma BM model. Lee et al. [218] showed that WP1066’s inhibition of STAT3 signaling in BM cells and brain endothelial cells resulted in a reduced tumor invasion capacity and downregulated angiogenesis/vascular permeability, respectively. However, present evidence is insufficient to confirm whether WP1066 works through anti-inflammatory STAT3 + astrocytes to intervene in the immunosuppressive TME. Two clinical trials (NCT04334863 and NCT01904123) are currently underway to evaluate the use of WP1066 in BM therapy.

Chen et al. [25] discovered that TNBC and NSCLC cells establish gap junctions with astrocytes through connexin 43 (Cx43) under the guidance of surface protocadherin 7 (PCDH7) (Fig. 4B). In this interaction, cGAMP, a cyclic nucleic acid derivative that was once regarded as a secondary messenger of innate antiviral immunity [219], moves into astrocytes along the channel and stimulates the STING signaling pathway. This dynamic results in the release of inflammatory factors, including IFNα and TNF, which, in turn, promote tumor growth, chemotherapy resistance and even the acquisition of FasL [25]. Current studies have demonstrated that both meclofenamate and tonabersat can significantly interfere with gap junctions, and when combined with carboplatin, they effectively suppress BM. They also show inhibitory potential on the accumulation of circulating tumor cells in the initial stage of breast cancer metastases [25, 220]. Encouragingly, a clinical trial (NCT02429570) of meclofenamate for BM therapy is currently underway.

Interestingly Zhao et al. [221] designed a delivery system called microenvironment-tailored micelles (T-M/siRNA) for BCBM. In this system, the TME specifically released paclitaxel to kill tumor cells, while the co-delivered siRNA silenced the gene encoding PCDH7. This effectively limited the cancer-astrocyte crosstalk mediated by intercellular channels and inhibited the STAT3 signal simulation, reducing the recruitment of metastases-promoting Iba1 + microglia/macrophages (Fig. 4B). Moreover, micelles could become a universal platform for carrying various siRNAs with TME modification functions, and they could be assembled into targeted structures for accurate delivery to different BM.

#### Microglia/macrophages

Another strategy aims to artificially impact resident or recruited myeloid immune cells, specifically, microglia/macrophages, in order to suppress BM (Table 3). Wu et al. [222] discovered a higher proportion of microglia with monocyte/macrophage infiltration might contribute to the increased BM burden in young breast cancer patients. They successfully reduced the number of reactively enhanced myelocytes in metastatic neuroinflammation by using the colony-stimulating factor 1 receptor (CSF1R) inhibitor PLX3397, effectively controlling BM in mouse models (Fig. 4C). Moreover, Klemm et al. [223] suggested that inhibiting the neuroinflammatory signaling pathway CSF2Rβ-STAT5, which promotes tumor recovery, using the drug AC4-130 could sustain tumor control and classical microglial activation when another CSF1R inhibitor, BLZ945, is used to intervene in tumor-associated microglia/macrophages (Fig. 4C). Besides the CSF1R inhibitor, Andreou et al. [30] reported a strategy where mannosylated clodronate liposomes reduce the infiltration of MRC1 + microglia/macrophages exhibiting an anti-inflammatory phenotype (metastases-promoting phenotype), considerably suppressing BM from mouse breast cancer.

Interestingly, Blazquez et al. [107] demonstrated that the PI3K inhibitor BKM120 reduced the brain invasion of malignant breast cells by managing metastases-promoting microglia/macrophages in the CNS, resulting in increased survival benefits. Wu et al. [224] found that tamoxifen inhibits the polarization of microglia into the M2 phenotype (an anti-inflammatory/protumor phenotype) mediated by the estrogen-STAT3 signaling pathway (Fig. 4D). M2 microglia secrete the cytokine CCL5 to promote metastases and overexpress signal regulatory protein α (SIRPα) combined with CD47 on the tumor surface to assist with immune evasion, hence effectively suppressing breast cancer BM in mice (Fig. 4D) [224]. These studies remind us that, as our understanding of the brain metastatic TME deepens, traditional tumor-targeting strategies such as PI3K inhibition and tamoxifen could have potential in immunotherapy.

Contrary to the strategy of inhibiting tumor-supporting myeloid cells, Benbenishty et al. [225] explored the approach of promoting the immune clearance activity of microglia. They found that the TLR 9 agonist, CpG-C, when administered systemically, could be taken up by microglia, which then got stimulated to more effectively kill and phagocytose tumor cells, especially at the initial stage of brain invasion (Fig. 4E).

In addition, Jin et al. [103] showed that fedratinib (JAK2 inhibitor) and tocilizumab (anti-IL6R) could also control BM from NSCLC. Although the researchers did not specifically investigate these drugs, their aim was to confirm that the IL6-JAK2-STAT3 signaling pathway plays a role in inducing the anti-inflammatory phenotype of microglia, which promotes BM cell colonization. This highlights that the JAK2-STAT3 pathway is not only important for astrocytes but also for microglia, and it may be a key target based on TME for future immunotherapy of BM.

#### Strategies with viruses and vaccines

Oncolytic viruses function primarily by activating antitumor immunity rather than by destroying infected cancer cells, which makes them an invaluable auxiliary tool in immunotherapy [109, 226]. She et al. [109]. discovered that oncolytic viruses stimulate the overexpression of the immune-enhancing gene IFITM1, thus greatly improving the efficacy of anti-PD-1 Given their mechanism of enhancing T cell immune recognition and microglial activation, it is anticipated that the immuno-inductive capabilities of oncolytic viruses could be combined with CAR-T cells or glial targeting drugs [199, 227].

Kanaya and colleagues developed a dual gene-edited/engineered stem cell-based delivery system for oncolytic viruses. By local injection, they successfully released viruses and immunomodulatory substances within the MBM, which resulted in notable therapeutic effects, while avoiding viral assault on the stem cells themselves [228]. Intriguingly, even though approved and investigational oncolytic virus therapies are typically directed at advanced melanoma [229], the study by Kanaya et al. [228] suggests that their findings could be potentially translated to other types of BM. This idea offers promising new avenues for future research into oncolytic viruses.

Cancer vaccines aim to stimulate the patient’s immune system to control tumor growth and eradicate diseases by administering selected tumor antigens and activating the body’s dendritic cells to maintain a strong immune response [230]. Even though evidence remains scant to advance the advocacy of dendritic cell-based antitumor vaccines as effective BM therapies, these vaccines nevertheless hold potential, particularly with a more nuanced understanding of the TME [97, 209].

## Conclusion and perspectives

BM cells do not indiscriminately wreak havoc on the brain environment; rather, they tactically exploit the inherent capability of glial cells to uphold BME stability. The tumor opportunistically leverages the dynamic interplay between proinflammatory and anti-inflammatory polarized brain cells, strategically shifting the equilibrium toward the anti-inflammatory end—an environment favorable for tumor survival. Subsequently, a BM fortress, marked by the TME safeguarded by immunosuppression barricades and underpinned by glial cells, is established. The examination of the BM microenvironment is pivotal to decoding the mechanics of immunotherapy and for the genesis of novel strategies. Presently, the primary types of immunotherapy approaches fall into two categories. The first seeks to alleviate immune suppression, including ICIs and interventions into tumor–brain cell interactions. The second aims to provide antitumor reinforcements, chiefly through the application of CAR-T cells. Additionally, the contribution of oncolytic viruses can supplement the effectiveness of immunotherapy.

Immunotherapy, originating from the ever-increasing understanding of the TME, naturally hits a ceiling due to the constraints of this very microenvironment. Specifically, hurdles such as the BBB and immunosuppressive brain cells impede the assault on intracranial lesions. Accordingly, the development of singular immune or immune pathway-targeting drugs reaches a limit. A more promising prospect might be the establishment of an intricate or comprehensive composite drug system harmonizing with the TME. Utilizing nanodelivery systems as a common platform for the co-loading of individualized units with immune, targeting, or chemotherapy drugs could be one developmental trajectory. Specifically, these nanodelivery systems can be controllably modified with target molecules capable of releasing ICIs, sensitive chemotherapy drugs or targeted drugs and RNA that interferes with tumor–glial cell interactions (particularly the STAT3 pathway). Of greater significance, evidence has emerged demonstrating that nanodelivery systems, including microenvironment-targeted micelles [221], radiation-induced cell-released microparticles [231] and liposomes [232], can successfully penetrate the BBB.

CAR-T cells will also benefit from the construction of nanodelivery systems: (i) the formulation of unique spaces, such as hydrogels, for the storage, release and potential activation assistance of CAR-T cells; (ii) the delivery of ICIs to bolster CAR-T cells, or even direct CAR mRNA delivery to prompt native T cells; and (iii) the use of physical response properties, such as light, heat, sound or magnetism, to assist CAR-T cells in better navigating and functioning within the TME [114, 190, 204, 207].

Despite its promise, it is essential to acknowledge that immunotherapy remains an evolving strategy, and its formal integration into clinical practice remains in future. On the one hand, while immunotherapy presents newfound hope for the treatment of recalcitrant BM, becoming a pivotal component of combined therapy and potentially supplementing or replacing surgical interventions, it may not eradicate the need for intracranial lesion resection. The prospective trial (NCT01628406) has demonstrated that smaller postoperative intracranial tumor residuals can significantly prolong patient survival when coupled with ICI therapy, particularly in those who have not previously received ICIs [233]. This approach garners the added benefit of reducing the burden of intracranial tumors. On the other hand, beyond laboratory breakthroughs, it is necessary to prioritize investigation into the synergistic application of immunotherapy with conventional or innovative physical therapies, including surgery, WBRT, SRS, LITT and FUS. Moreover, localized applications warrant exploration at this stage [50], given their potential to circumvent the permeability issue of the BBB, significantly enhance delivery efficiency, address clinical challenges related to positive surgical margins and reasonably control systemic toxicity.

## Data Availability

Not applicable.

## Abbreviations

BM: Brain metastases
BME: Brain microenvironment
TME: Tumor microenvironment
CAR-T cells: Chimeric antigen receptor T cells
SEER: Surveillance epidemiology and end result
WHO: World Health Organization
NSCLC: Non-small cell lung cancer
SCLC: Small cell lung cancer
BCBM: Breast cancer brain metastases
MBM: Melanoma brain metastases
CNS: Central nervous system
BBB: Blood–brain barrier
MICs: Metastases-initiating cells
CTC: Circulating tumor cell
EMT: Epithelial–mesenchymal transition
MET: Mesenchymal–epithelial transition
DAMP: Damage-associated molecular patterns
PAMP: Pathogen-associated molecular patterns
WBRT: Whole-brain radiotherapy
SRS: Stereotactic radiotherapy
LITT: Laser interstitial thermotherapy
FUS: Focused ultrasound
VEGF: Vascular endothelial growth factor
HIF1α: Hypoxia-inducible factor 1α
BTB: Brain tumor barrier
MMP: Matrix metalloproteinase
S1P3: Sphingosine-1 phosphate receptor 3
BLBC: Basal-like breast cancer
TNBC: Triple-negative breast cancer
HLA-A: Human leukocyte antigen-A
CSF: Cerebrospinal fluid
TILs: Tumor-infiltrating lymphocytes
TGLI1: Truncated glioma-associated oncogene homolog 1
MCP-1: Monocyte chemoattractant protein-1
DAG: Dystroglycan
DTC: Dissected tumor cell
YAP: Yes-associated protein
FOXA2: Forkhead box A2
EVs: Extracellular vesicles
CXCL10: C-X-C motif chemokine 10
CNTF: Ciliary neurotropic factor
CNTFRα: Ciliary neurotropic factor receptor alpha
cGAMP: Cyclic GMP-AMP
CX43: Connexin 43
PCDH7: Protocadherin 7
STING: Stimulator of interferon genes
INFα: Interferon alpha
TNF: Tumor necrosis factor
STAT: Signal transducer and activator of transcription
NF-κB: Nuclear factor kappa-B
TGF: Transforming growth factor
IL: Interleukin
CCL: C-C motif chemokine ligand
JAG1: Jagged1
HES5: Hes family bHLH transcription factor 5
AA: Arachidonic acid
PPARγ: Peroxisome proliferator-activated receptor gamma
EGF: Epidermal growth factor
JAK2: Janus kinase 2
MIF: Macrophage migration inhibitory factor
SIRPα: Signal regulatory protein alpha
IL–6R: Interleukin-6 receptor
gp130: Glycoprotein 130
PI3K: Phosphoinositide 3-kinase
C/EBPβ: C/CAAT enhancer binding protein beta
ANXA1: Annexin-A1
FPR: Formyl peptide receptor
ERK: Extracellular signal-regulated kinase
CXCL10: C-X-C motif chemokine ligand 10
CXCR3: C-X-C motif chemokine receptor 3
PD-1: Programmed death 1
PD-L1: Programmed death ligand 1
APCs: Antigen-presenting cells
ICI: Immune checkpoint inhibitors
RCC: Renal cell carcinoma
RN: Radiation necrosis
LAG-3: Lymphocyte-activating gene-3
CTLA-4: Cytotoxic T lymphocyte-associated protein-4
PR: Partial response
OS: Overall survival
mOS: Median overall survival
PFS: Progression-free survival
mPFS: Median progression-free survival
AEs: Adverse events
TCR: T cell receptor
TAAs: Tumor-associated antigens
VH: Variable heavy chain
VL: Variable light chain
NFAT: Nuclear factor of activated T cells
FDA: Food and drug administration
ECM: Extracellular matrix
MWA: Microwave ablation
MDSCs: Myeloid-derived suppressor cells
TAMs: Tumor-associated macrophages
Tregs: Regulatory T cells
Bregs: Regulatory B cells
